# Mechanism by which low-intensity focused ultrasound promotes angiogenesis and neurogenesis after traumatic brain injury in a rat model via the OXA/MAPK signaling pathway

**DOI:** 10.4103/NRR.NRR-D-24-01153

**Published:** 2025-04-29

**Authors:** Bingkai Ren, Junwei Kang, Peng Yao, Lianghua Huang, Yan Wang, Yang Bai, Zhen Feng

**Affiliations:** 1 Affiliated Rehabilitation Hospital, Jiangxi Medical College, Nanchang University, Nanchang, Jiangxi Province, China; 2 Rehabilitation Medicine Clinical Research Center of Jiangxi Province, Nanchang, Jiangxi Province, China; 3 Key Laboratory of Jiangxi Provincial Health Commission for DOC Rehabilitation, Nanchang, Jiangxi Province, China; 4 Department of Rehabilitation Medicine, The First Affiliated Hospital of Nanchang University, Nanchang, Jiangxi Province, China

**Keywords:** angiogenesis, blood–brain barrier, low-intensity focused ultrasound, MAPK signaling pathway, nerve repair, neurogenesis, neurological function, orexin-A, OX1R, traumatic brain injury

## Abstract

Low-intensity focused ultrasound is a type of ultrasound that primarily relies on cavitation and mechanical effects. It is non-invasive, transient, and well tolerated. Previous studies have confirmed that low-intensity focused ultrasound can reduce neuroinflammation after traumatic brain injury and exert neuroprotective effects. However, whether it can also induce angiogenesis and neurogenesis in the brain, as well as the underlying mechanisms, remains unclear. In this preclinical study, a rat model of traumatic brain injury was established using a controlled cortical impact device. The rats were then received 14 days of low-intensity focused ultrasound treatment targeting the thalamus. The results showed that low-intensity focused ultrasound effectively reduced cerebral edema and mitigated blood–brain barrier damage in rats with traumatic brain injury, leading to improved neurological function. Further investigation showed that low-intensity focused ultrasound significantly un-regulated Orexin-A/Orexin-A receptor 1 expression, and intraperitoneal administration of the Orexin-A receptor 1 inhibitor SB334867 prevented the neuroprotective effects of low-intensity focused ultrasound. Subsequent transcriptome sequencing revealed that low-intensity focused ultrasound activated the MAPK signaling pathway. Finally, in an *in vitro* cell injury model created using tumor necrosis factor-alpha, low-intensity focused ultrasound enhanced endothelial cell migration, stimulated angiogenesis, and supported hippocampal neuron migration and growth. Moreover, the MAPK signaling pathway inhibitor LY3214996 suppressed these effects. Taken together, our findings suggest that low-intensity focused ultrasound enhances angiogenesis and neurogenesis and improves neurological function following traumatic brain injury by regulating the expression of Orexin-A/Orexin-A receptor 1, which activates the MAPK signaling pathway.

## Graphical abstract

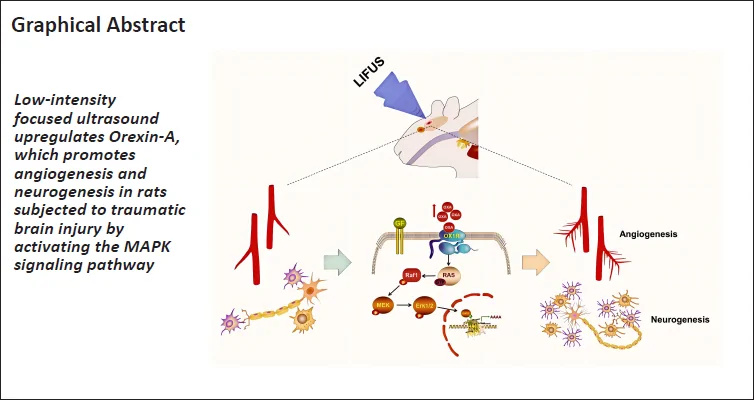

## Introduction

With an annual incidence exceeding 50 million cases, traumatic brain injury (TBI) remains a leading cause of neurological impairment and mortality worldwide (Dewan et al., 2019; Ding et al., 2022; Maiese et al., 2023) and a significant factor in the development of lasting disabilities among adolescents (Langlois et al., 2006). TBI involves both primary and secondary injury. Primary injury, caused by direct external forces, includes brain stem injury, cerebral contusion, diffuse axonal injury, and laceration, all of which lead to irreversible neurological damage. By contrast, secondary injury refers to the gradual deterioration of nerves resulting from successive pathophysiological changes following trauma, such as cerebral ischemia, brain edema, and neuronal apoptosis. Treating these secondary injuries is crucial because, unlike the primary injury, they can be alleviated or reversed (Robinson, 2021).

Axon regeneration and neurogenesis are pivotal in replenishing lost neurons and reconstructing neural regulatory networks within the injured brain, facilitating neurological recovery post-TBI (He et al., 2020; Redell et al., 2020; Wang et al., 2025). The neurological deficits that ensue after TBI are partly attributed to the inherent limitations of neurogenesis and axon regeneration (Jarrahi et al., 2020). Furthermore, the hippocampus is particularly susceptible to subsequent cascades of damage, resulting in a considerable number of newly formed immature neurons undergoing apoptosis during the initial stages following TBI, thereby hindering neurogenesis in the dentate gyrus (Rola et al., 2006; Gao et al., 2008). Although the exact mechanisms underlying spontaneous brain recovery are still unknown, newly generated neurons within the injured brain are believed to replace lost neurons (Magavi et al., 2000). Disrupting this process can hinder natural regeneration and is linked to cognitive impairment (Blaiss et al., 2011). Enhanced neural recovery is positively associated with the degree of neurogenesis (Liu et al., 2007; Schäbitz et al., 2007; Meng et al., 2011), with specific cellular signaling pathways playing a crucial role in regulating neurogenesis (Cai et al., 2012). Therefore, promoting neurogenesis and axon regeneration holds considerable promise for improving recovery from TBI (Hu et al., 2023).

The cerebral vascular network is essential for sustaining brain metabolism and function. TBI commonly leads to cerebrovascular dysfunction (Jullienne et al., 2016), greatly contributing to secondary brain damage. During the acute phase of TBI, there is an increase in blood–brain barrier (BBB) permeability due to damage to cerebral vascular integrity (Zhang et al., 2025). This disruption leads to vasogenic cerebral edema, increasing intracranial pressure, exacerbating brain tissue swelling, and decreasing perfusion pressure (Guo et al., 2021). In the subsequent acute and subacute phases, neovascularization within the penumbra region enhances oxygen and nutrient delivery to the affected tissue and improves brain function (Ma et al., 2020; Zhang et al., 2021; Liu et al., 2022a). Establishing robust collateral circulation early in the injury process is crucial for optimal brain recovery. Consequently, promoting angiogenesis has emerged as a promising therapeutic strategy for TBI.

The neuropeptide orexin-A (OXA) is synthesized and secreted by the lateral hypothalamic area, projecting to the prefrontal cortex. The orexin system, through its receptors OX1R and OX2R, governs a range of physiological processes such as sleep–wake cycles, feeding behavior, energy metabolism, and neuroendocrine activity (Li and de Lecea, 2020). OXA attenuates neuronal degeneration and prevents cell death (Adamantidis et al., 2007; Shirasaka et al., 2011). We previously showed that OXA effectively inhibits ferroptosis following TBI in rats, suggesting that it plays a neuroprotective role (Kang et al., 2024). Cell-based experiments have shown that OXA is a novel pro-angiogenic peptide (Kim et al., 2010). However, whether OXA can promote angiogenesis and improve neurological function after TBI remains poorly understood.

Recent studies have validated the angiogenic and neurogenic effects of low-intensity focused ultrasound (LIFUS) (Shindo et al., 2016; de Lucas et al., 2020). As a non-invasive treatment option, LIFUS is associated with a minimal risk of complications compared with other rehabilitation modalities. Notably, this therapy does not require costly equipment, yet it enables precise targeting of specific brain regions for therapeutic intervention. Furthermore, it boasts lower treatment expenses and enhanced feasibility, and thus holds significant potential for clinical translation (Bubrick et al., 2022; Attali et al., 2023). *In vitro* studies have shown that LIFUS stimulates astrocytes to release of neurotrophic factors such as brain-derived neurotrophic factor and vascular endothelial growth factor (VEGF) (Liu et al., 2017; Chen et al., 2019). *In vivo* studies have shown that LIFUS promotes angiogenesis and synaptogenesis, resulting in elevated cerebral blood flow, reduced ischemic brain area, and improved neurological function (Karmacharya et al., 2015; Ichijo et al., 2021; Deng et al., 2022; Qi et al., 2024). Furthermore, studies have indicated that LIFUS can enhance consciousness levels in patients with TBI who are in a coma, leading to substantial improvements in Coma Recovery Scale-Revised scores following stimulation. The current study builds upon our prior work pioneering the application of LIFUS for treating acute TBI in a rat model. We previously showed that LIFUS significantly upregulates OXA expression, resulting in reduced neuroinflammation and exerting a neuroprotective effect (Huang et al., 2022). On the basis of these findings, we hypothesized that LIFUS enhances neurological function in brain-injured rats by promoting angiogenesis and neurogenesis through OXA. However, the precise mechanisms underlying the beneficial effects of LIFUS therapy on TBI and the optimal targets for stimulation remained to be elucidated. Herein, we investigated the capacity of LIFUS to enhance recovery from post-TBI neurotrauma by inducing cerebral angiogenesis and neurogenesis.

## Methods

### Animals

The Ethics Committee of The First Affiliated Hospital of Nanchang University approved all animal experiments (No. CDYFY-IACUC-202306QR040). All procedures were performed in accordance with the National Institutes of Health Guide for the Care and Use of Laboratory Animals (National Research Council, 2011). One hundred and fifty 6-week-old male Sprague–Dawley rats of specific pathogen–free grade were used in this study. The rats, weighing between 250 and 300 g, were sourced from Zhejiang Weitong Lihua Experimental Animal Technology Co., Ltd. (License No. SCXK (Zhe) 2019-0001) to ensure experimental consistency. They were maintained under standardized conditions, including a controlled environment at 25°C, a 12/12-hour light/dark cycle, and access to food and water *ad libitum*. The animal experiments for LIFUS treatment of TBI were divided into two stages (detailed experimental procedures are shown in **Additional Figure 1A**). In the first stage, 48 rats were randomly divided into four groups: the sham group (*n* = 12), CCI group (*n* = 12), CCI + LIFUS group (*n* = 12), and CCI + sham-LIFUS group (*n* = 12). In the second stage, 60 rats were randomly divided into five groups: the shamgroup (*n* = 12), CCI group (*n* = 12), CCI + LIFUS group (*n* = 12), CCI + sham-LIFUS group (*n* = 12), and CCI + LIFUS + SB334867 group (*n* = 12). For the *in vivo* Matrigel plug experiment (detailed experimental procedures are shown in **Additional Figure 1B**), in the first stage, 16 rats were randomly divided into four groups: the sham group (*n* = 4), tumor necrosis factor-alpha (TNF-α) group (*n* = 4), TNF-α + OX-A group (*n* = 4), and TNF-α + OX-A + normal saline group (*n* = 4). In the second stage, another 16 rats were randomly divided into four groups: the sham group (*n* = 4), TNF-α group (*n* = 4), TNF-α + OX-A group (*n* = 4), and TNF-α + OX-A + LY3214996 group (*n* = 4). A total of 10 rats died during the experiments. Experimental data were collected by a researcher who was blinded to the group assignments.

### Creating a rat model traumatic brain injury

A controlled cortical impact was used to induce TBI in rats, as previously described (Ren et al., 2024). Anesthesia was induced by inhalation of 4% isoflurane (RWD, Shenzhen, China) and maintained by oral administration of 2.5% isoflurane through a face mask. Experimental rats were immobilized within a rat stereotaxic apparatus (RWD). After preparing and disinfecting the surgical site, a scalp incision was made, followed by dissection of the subcutaneous tissue and periosteum. A circular craniectomy with a diameter of 5.0 mm was performed on the left parietal cortex (from the bregma: 0.5 to 4.5 mm anterior-posterior and 0 to 5 mm medial-lateral, as per Sun et al. (2023), ensuring that no tissue was damaged. The controlled cortical impact device (YHCI99, Wuhan Yihong Technology Co., Ltd., Wuhan, Hubei Province, China) was then precisely positioned over the craniectomy site and equipped with a 4.0-mm flat impactor tip. Moderate TBI was induced using the controlled cortical impact device, operating at a velocity of 4 m/s, with an impact depth of 2.8 mm and a dwell time of 150 ms.

### Low-intensity focused ultrasound

LIFUS treatment was applied as we described previously (Huang et al., 2022). The ultrasonic transducer (V301-SU; Olympus, Japan) received the ultrasonic pulse signal from the ultrasound generator (NTK-UNS-01 system, Jiangxi Brain Regulation, China). This transducer, which has a focal length of 33 mm, was equipped with a replaceable 3D-printed conical collimator. LIFUS treatment was initiated on the first day after TBI. The rat’s head was securely held in place, and the focal distance of the ultrasonic probe was adjusted using quasi-actuators to target ultrasound waves at the hypothalamus region. The ultrasonic wave had a frequency of 500 Hz, a duty cycle of 5%, a pulse repetition frequency of 250 Hz, and a total duration of 400 ms. This configuration produced a spatial-peak temporal-average intensity of 518 mW/cm² in degassed water. For sham LIFUS, the ultrasound machine was operated normally, but the probe was positioned away from the skull to prevent ultrasonic stimulation. Each rat underwent a daily treatment session lasting 20 minutes for a total of 14 days.

### Administration of intraperitoneal injections

Bromodeoxyuridine (BrdU; Solarbio, Beijing, China, Cat# 59-14-3) was dissolved in normal saline to a concentration of 10 mg/mL for *in vivo* experiments. BrdU was administered intraperitoneally at a dosage of 50 mg/kg, twice daily. SB334867 (Orexin-A receptor 1 inhibitor; MedChemExpress, Newark, NJ, USA, HY-10895) was dissolved in dimethyl sulfoxide and administered intraperitoneally at a dose of 30 mg/kg once daily to the LIFUS + SB334867 group. As a control, 1 mL of 5% dimethyl sulfoxide was injected. The drugs were administered post-brain injury and continued at these dosages until the time of death.

### Modified Neurological Severity Scale

Motor, sensory, balance, and reflex functions were evaluated using the modified Neurological Severity Score (mNSS), a comprehensive assessment tool. The assessment protocol adhered to the methodologies described in previous studies (Tang et al., 2020; Huang et al., 2022). Scores were categorized as follows: 1 to 6 for mild impairment, 7 to 12 for moderate impairment, and 13 to 18 for severe impairment. mNSS assessments were conducted at baseline and on days 1, 3, 7, and 14 post-TBI. All neurobehavioral evaluations were performed by two researchers who were blinded to the group allocations.

### Morris water maze test

The Morris water maze is a widely used experimental tool for evaluating spatial learning and memory in rodents (Liu et al., 2022b). The experimental protocol consisted of training and testing phases. During training, the subject was released into the pool from a randomly selected starting location opposite the platform. Upon reaching the platform, the subject remained there for 5 seconds. If the animal was unsuccessful in finding the platform within 90 seconds, it was gently guided to the platform and allowed to stay there for 15 seconds. In the testing phase, the rat was introduced into a pool without a platform at its initial starting point. The duration spent in the designated quadrant and the frequency of crossings over the platform within a 120-second interval were recorded. VisuTrack software (Softmaze, Shanghai, China) was used to quantify escape latency during training, as well as target quadrant occupancy and platform crossings during the testing phase.

### Brain water content

Brain water content was determined as previously described (Tang et al., 2020; Huang et al., 2022). Rats were anesthetized using Zoletil 50 (Virbac, Nice, France) and euthanized by cardiac perfusion, followed by rapid brain extraction. The brain was then cut along the midline into left and right hemispheres using a surgical blade. Prior to analysis, the samples were weighed using an electronic balance (Changxie Electronic Products Factory, Dongguan, Guangdong, China, CX-12000) to determine their wet weight. The samples were subsequently dried in an oven set at 100°C for 24 hours. After drying, the samples were reweighed to obtain their dry weight. To calculate brain water content as a percentage, the following formula was used: water content (%) = 100% × (wet weight – dry weight)/wet weight.

### Blood–brain barrier permeability

At 7 days post-TBI, 2% Evans blue (4 mL/kg; Yuanye Bio, Shanghai, China) was injected via the tail vein. Four hours later, residual dye in the vasculature was removed by perfusing the left ventricle with normal saline. The left cerebral hemisphere of the rats was then quickly removed, weighed, and homogenized in 0.5 mL dimethyl sulfoxide per 50 mg of tissue. The homogenate was incubated for 24 hours at 60°C, followed by centrifugation at 1000 × *g* for 5 minutes. The concentration of Evans blue in the supernatant was determined by measuring the optical density at 620 nm using a multifunctional microplate reader (Thermofisher, Waltham, MA, USA, Varioskan LUX) and a pre-established standard curve.

### Transmission electron microscopy

A standardized protocol was used to examine microvascular structures within rat brain tissue by transmission electron microscopy. First, a 1-mm^3^ section of rat cortical tissue was immersed in electron microscopy fixative for 1 hour. The tissue was then post-fixed in 1% osmium tetroxide for 2 hours in the dark. Dehydration was achieved through a graded ethanol series, ranging from 30% to 95%. Following dehydration, the tissue was infiltrated with and embedded in a mixture of acetone and 812 embedding medium. Ultrathin sections, measuring 60 nm in thickness, were obtained using an ultramicrotome (Leica UC7, Leica, Vizsla, Germany) and collected on Formvar-coated 150-mesh copper grids. To enhance contrast, the sections were stained with 2% uranyl acetate in ethanol for 8 minutes in the dark, followed by three washes in 70% ethanol and ultrapure water. The sections were then stained with 2.6% lead citrate for 8 minutes and rinsed three times with ultrapure water. After overnight air-drying, the sections were examined under a transmission electron microscope (HT7700, Hitachi, Tokyo, Japan). Image acquisition and subsequent analysis of caveolae density were performed using ImageJ software (v1.54d, National Institutes of Health, Bethesda, MD, USA; Schneider et al., 2012).

### Bioinformatic analysis

Rats in both the TBI and LIFUS treatment groups were subjected to cardiac perfusion on day 14 post-TBI, and brain tissue samples were collected for subsequent transcriptome sequencing. The transcriptome sequencing was conducted by Suzhou Biotechnology Co., Ltd. Gene expression data were analyzed, and differential gene expression between groups was analyzed using a screening threshold of |log2 fold change| ≥ 1 and *P* < 0.05. Volcano plots were then generated using R Studio statistical software to visualize the differentially expressed genes. Additionally, Kyoto Encyclopedia of Genes and Genomes (KEGG) pathway enrichment analysis was conducted to identify significantly overrepresented pathways.

### Hematoxylin and eosin staining

After paraffin embedding and sectioning, brain tissue sections were progressively dehydrated using ethanol solutions of increasing concentrations. The brain tissue sections were then subjected to hematoxylin and eosin (HE) staining (Servicebio, Wuhan, China, G1076). The staining protocol included the following steps: deparaffinization, rehydration through a graded ethanol series, hematoxylin staining for 3–5 minutes, differentiation, bluing, eosin staining for 5 minutes, dehydration, clearing, and coverslipping. Finally, the stained sections were imaged using an optical microscope (NIKON ECLIPSE E100, Tokyo, Japan).

### Terminal deoxynucleotidyl transferase-mediated dUTP nick end labeling

Apoptotic cells in paraffin-embedded cortical tissue sections were identified by TUNEL assay (Servicebio, G1502). The Cy3-labeled fluorescein provided with the TUNEL kit stained apoptotic nuclei red, while 4′,6-diamidino-2′-phenylindole was used to counterstain normal nuclei blue. The apoptotic index was calculated by dividing the number of TUNEL-positive nuclei by the total number of nuclei. Images were acquired using a fluorescence microscope (NIKON ECLIPSE C1), and subsequent analysis was conducted with ImageJ software by an independent observer. At least three stained slides were evaluated for each rat.

### Immunohistochemistry staining

Immunohistochemical analysis was conducted on rat brain tissue to assess blood vessel density surrounding the injury site. Standard immunohistochemistry protocols were followed for dewaxing, rehydration, and antigen retrieval. Endogenous peroxidase activity was neutralized by treating the sections with a 3% hydrogen peroxide solution, and non-specific binding was prevented by incubating the sections with 3% bovine serum albumin in phosphate-buffered saline. The sections were then incubated overnight at 4°C in a humidified chamber with primary antibodies. Following three washes with PBS (pH 7.4), the sections were incubated for 50 minutes at room temperature with a secondary antibody. The details of the primary and secondary antibodies used are provided in **Table 1**. After additional washes with PBS, diaminobenzidine (Servicebio, G1212) was applied. Once sealed, the slides were examined using an optical microscope.

**Table 1:** Primary and secondary antibodies used in this study

Antibody	Host	Dilution	Vendor	Catalog number	RRID	Molecular weight (kDa)	Application
**Primary antibody**							
CD31	Rabbit	1:1000 for WB; 1:500 for IF and IHC	Abcam	AB281583	AB_3096925	125	WB, IHC, IF
VEGFA	Rabbit	1:1000	Abcam	AB214424	AB_3064726	40	WB
Ang1	Rabbit	1:1000	Affinity	AF5184	AB_2837670	57	WB
Orexin-A	Rabbit	1:1000	Bioss	bs-15509R	AB_3665856	13	WB
OX1R	Rabbit	1:1000 for WB; 1:200 for IF	Huabio	ER1914-44	AB_3665862	48	WB, IF
Occludin	Rabbit	1:1000	Abcam	AB216327	AB_2737295	59	WB
VE-Cadherin	Rabbit	1:1000	CST	2500	AB_10839118	130–140	WB
TJP1	Rabbit	1:1000	Boster	PB9234	AB_3082026	220	WB
NeuN	Rabbit	1:1000 for WB; 1:200 for IF	CST	24307	AB_2651140	46–55	WB, IF
BrdU	Rat	1:200	Abcam	AB6326	AB_305426		IF
DCX	Rabbit	1:200	Abcam	AB207175	AB_2894710	40	IF
Bax	Rabbit	1:1000	CST	2772	AB_10695870	20	WB
Bcl-xl	Rabbit	1:1000	CST	2764	AB_2228008	30	WB
MAP2	Rabbit	1:1000 for WB; 1:200 for IF	CST	4542	AB_10693782	75	WB, IF
GAP43	Rabbit	1:1000 for WB; 1:200 for IF	CST	5307	AB_10622185	43	WB, IF
Ras	Rabbit	1:1000	CST	67648	AB_2910195	21	WB
Raf	Rabbit	1:1000	Boster	BM4108	AB_3665863	73	WB
p-Raf	Rabbit	1:1000	Boster	BM4780	AB_3665864	73	WB
MEK	Rabbit	1:1000	Boster	BM4055	AB_3665865	43	WB
p-MEK	Rabbit	1:1000	Boster	BM4690	AB_3665866	43	WB
ERK	Rabbit	1:1000	CST	4695	AB_390779	44 and 42	WB
p-ERK	Rabbit	1:2000 for WB; 1:200 for IF	CST	4370	AB_2315112	44 and 42	WB, IF
GAPDH	Rabbit	1:5000	Proteintech	10494-1-AP1	AB_2263076	36	WB
GAPDH	Mouse	1:5000	Proteintech	60004-1-Ig	AB_2107436	36	WB
β-Actin	Mouse	1:5000	Proteintech	66009-1-Ig	AB_2687938	42	WB
β-Tubulin	Rabbit	1:1000	Immunoway	YT4780	AB_3665860	55	WB
**Secondary antibody**							
HRP conjugated goat anti-rabbit IgG	Goat	1:500	Servicebio	GB23303	AB_2811189		IF
HRP conjugated goat anti-rat IgG	Goat	1:500	Servicebio	GB23302	AB_2928139		IF
HRP conjugated goat anti-rabbit IgG	Goat	1:10000	ZSGB-BIO	ZB-2301	AB_2747412		WB
HRP conjugated goat anti-mouse IgG	Goat	1:10000	ZSGB-BIO	ZB-2305	AB_2747415		WB

Ang-1: Angiopoietin-1; Bax: BCL-2-associated X protein; Bcl-xl: B-cell lymphoma-extra large; BrdU: 5-bromo-2'-deoxyuridine; CD31/PECAM-1: platelet endothelial cell adhesion molecule-1; CST: Cell Signaling Technology; DCX: doublecortin; ERK: extracellular regulated protein kinases; GAP43: growth associated protein 43; GAPDH: glyceraldehyde-3-phosphate dehydrogenase; HRP: horseradish peroxidase; IF: immunofluorescence; IgG: immunoglobulin G; IHC: immunohistochemistry; MEK: mitogen-activated extracellular signal-regulated kinase; MAP2: microtubule-associated protein-2; NeuN: neuronal nuclear antigen; OX1R: orexin receptor 1; P-ERK: phosphorylated extracellular regulated protein kinases; p-MEK: phosphorylated mitogen-activated extracellular signal-regulated kinase; p-Raf: phosphorylated rapidly accelerated fibrosarcoma; Raf: rapidly accelerated fibrosarcoma; Ras: rat sarcoma; TJP1: ZO1 tight junction protein; VEGF: vascular endothelial growth factor; WB: western blotting.

### Immunofluorescence staining

After dewaxing and hydration, rat brain tissue and Matrigel sections underwent antigen retrieval, after which the sections were immersed in a 3% bovine serum albumin solution for 30 minutes to minimize non-specific binding. The sections were then incubated overnight at 4°C with primary antibodies. The following day, the sections were incubated with a Cy3-conjugated secondary antibody for 50 minutes at room temperature protected from light. For the fluorescence double-labeling experiment, an HRP-conjugated secondary antibody was applied, followed by a 50-minute incubation at room temperature. Next, the corresponding TSA dyes (iF488, Servicebio, Cat# G1231; iF555, Servicebio, Cat# G1233) were added, and the sections were incubated in the dark at room temperature for an additional 10 minutes. The details of the primary and secondary antibodies are provided in **Table 1**. Subsequently, nuclear staining was performed using DAPI for 8 minutes at room temperature (30–40°C), also shielded from light. Autofluorescence quenching was achieved by incubating the sections with a dedicated reagent for 5 minutes. After rinsing the sections with water for 10 minutes, they were mounted using an anti-fading medium compatible with fluorescence quenching. Finally, images were acquired using a fluorescence microscope.

### Western blotting

Following the manufacturer’s protocol, protein extraction was performed by RIPA lysis buffer (APExBIO, Shanghai, China, K1020), and the total protein concentration was measured using a bicinchoninic acid protein quantification kit (Solarbio, PC0020). Samples containing equal amounts of protein were then subjected to polyacrylamide gel electrophoresis and rapidly transferred onto a nitrocellulose membrane. To prevent non-specific binding, the membranes were incubated in 5% non-fat milk at room temperature for 2 hours. Then, the membranes were incubated with primary antibodies targeting various proteins overnight at 4°C. Following three washes of 15 minutes each with Tris-buffered saline containing Tween 20, the membranes were incubated with secondary antibodies for 1 hour at room temperature. The details of the primary and secondary antibodies used are provided in **Table 1**. Visualization of protein bands was achieved through enhanced chemiluminescence (Uelandy, Jiangsu, China), and band intensities were quantified using ImageJ software. The relative expression level of target protein was normalized by the corresponding internal reference.

### Quantitative polymerase chain reaction

Total RNA was extracted from rat brain tissues using Trizol reagent (Invitrogen, Carlsbad, CA, USA), followed by complementary DNA (cDNA) synthesis using a PrimeScript RT kit (TransGen Biotech, Beijing, China). Quantitative polymerase chain reaction (qPCR) analysis was conducted using SYBR Green PCR Master Mix (TransGen Biotech, Beijing, China) on a CFX96 Real-Time System (Bio-Rad, Hercules, CA, USA), following the manufacturer’s guidelines. The internal control gene used for normalization was glyceraldehyde-3-phosphate dehydrogenase (GAPDH). Target gene mRNA expression levels were normalized using the 2^–ΔΔCt^ method (Munshi et al., 2025). Detailed primer sequences are provided in **Table 2**.

**Table 2:** Polymerase chain reaction primers used in this study

Gene	Accession number	Sequence (5'–3')	Amplication length (bp)
*OXR1*	NM_013064.2	Forward primer: GAG TGT TTG GGA TGT TTC GC	143
		Reverse primer: GAA CTG CTC CCG AAA TTT GC	
*R-GAPDH*	NM_017008.4	Forward primer: CTG GAG AAA CCT GCC AAG TAT G	138
		Reverse primer: GGT GGA AGA ATG GGA GTT GCT	

### Cell culture and drug administration

HCMEC/D3 cells (iCell-h070, RRID: CVCL_U985) were obtained from iCell Bioscience (Shanghai, China) and maintained in an endothelial cell–specific medium (iCell-h070-001b). H19-7 cells (lot No. HTX4087, RRID: CVCL_H781) were sourced from Otwo Biotech (Shenzhen, China) and cultured in high-glucose DMEM supplemented with 10% fetal bovine serum. Both cell lines were grown in a humidified incubator at 37°C with 5% CO_2_ and 95% air. To investigate the effects of OXA (MedChemExpress, Newark, NJ, USA, HY-106224) on cellular responses induced by TNF-α (Abbkine, Wuhan, China, PRP1013) and to explore potential underlying mechanisms, the cells were treated with varying concentrations of TNF-α (10 ng/mL) and OXA (0.1, 0.5, and 1 μM). Additionally, the extracellular regulated protein kinase 1/2 (ERK1/2) signaling pathway inhibitor LY3214996 (MedChemExpress, HY-101494) was used at a concentration of 1 μM. Detailed experimental procedures are shown in **Additional Figure 1C** and **D**.

### Wound healing assay

HCMEC/D3 and H19-7 cells were seeded into 6-well culture plates and grown to confluence in specialized media. A linear scratch was created in the cell monolayer using a sterile 200 μL pipette tip, followed by three washes with PBS to remove any cellular debris. The cells were then incubated for 24 hours in medium supplemented with 1% fetal bovine serum, containing OXA, TNF-α, LY3214996, or their combinations. Cellular migration in three randomly selected fields was assessed at 0, 12, and 24 hours using an optical microscope. The percentage of gap closure was calculated using the following formula: gap closure percentage = 100 × (*W*_0_ – *W*_24_ or *W*_12_)/*W*_0_.

### Tube formation assay

Matrigel (BD, Corning Matrigel, Corning, NY, USA, 354262) was diluted to 6 mg/mL, and 150 μL was added to each well of a 48-well culture plate and allowed to solidify overnight at 37°C. Next, 60,000 HCMEC/D3 cells were seeded into each well in 300 μL of serum-free medium. The culture medium was supplemented with varying concentrations of TNF-α (10 ng/mL), OXA (0.1, 0.5, and 1 μM), LY3214996 (1 μM), or combinations thereof. After a 6-hour incubation at 37°C, tubular structures were visualized using an optical microscope and quantified using ImageJ software.

### *In vivo* rat Matrigel plug assay

Matrigel was combined with cell suspensions containing OXA, TNF-α, LY3214996, normal saline, or their combinations to achieve a final concentration of 7 mg/mL after thorough mixing. The specific compositions were as follows: TNF-α at 10 ng/mL; TO2 (TNF-α at 10 ng/mL combined with Orexin-A at 0.5 μM); TO2LY (TNF-α at 10 ng/mL combined with Orexin-A at 0.5 μM and LY3214996 at 1 μM); and TNC (TNF-α at 10 ng/mL combined with normal saline). The mixtures were subcutaneously injected into the axillary regions of the rats, and 7 days later the rats were euthanized. The Matrigel plugs were then excised, fixed in 4% paraformaldehyde, embedded in paraffin, and sectioned for analysis. Hematoxylin-eosin staining and CD31 immunofluorescence staining were performed on the sections. Angiogenesis was quantitatively assessed by measuring fluorescence intensity using ImageJ software.

### Statistical analysis

No prespecified statistical sample size was established for this study. However, the sample sizes used per group are consistent with those reported in previous studies (Tang et al., 2020; Sakas et al., 2023). Neurological deficits were evaluated by blinded assessors, and all relevant quantitative results were analyzed by team members who were unaware of the group assignments, using GraphPad Prism (version 10.1.2 for Windows, GraphPad Software, Boston, MA, USA, www.graphpad.com). To assess differences among groups, one-way analysis of variance (ANOVA) was conducted for normally distributed data, followed by Tukey’s *post hoc* test. For data that did not conform to a normal distribution, the Kruskal–Wallis test was used. Statistical significance was defined as a *P*-value of less than 0.05.

## Results

### Potential of low-intensity focused ultrasound to promote angiogenesis and neurogenesis after traumatic brain injury

**Figure 1:**
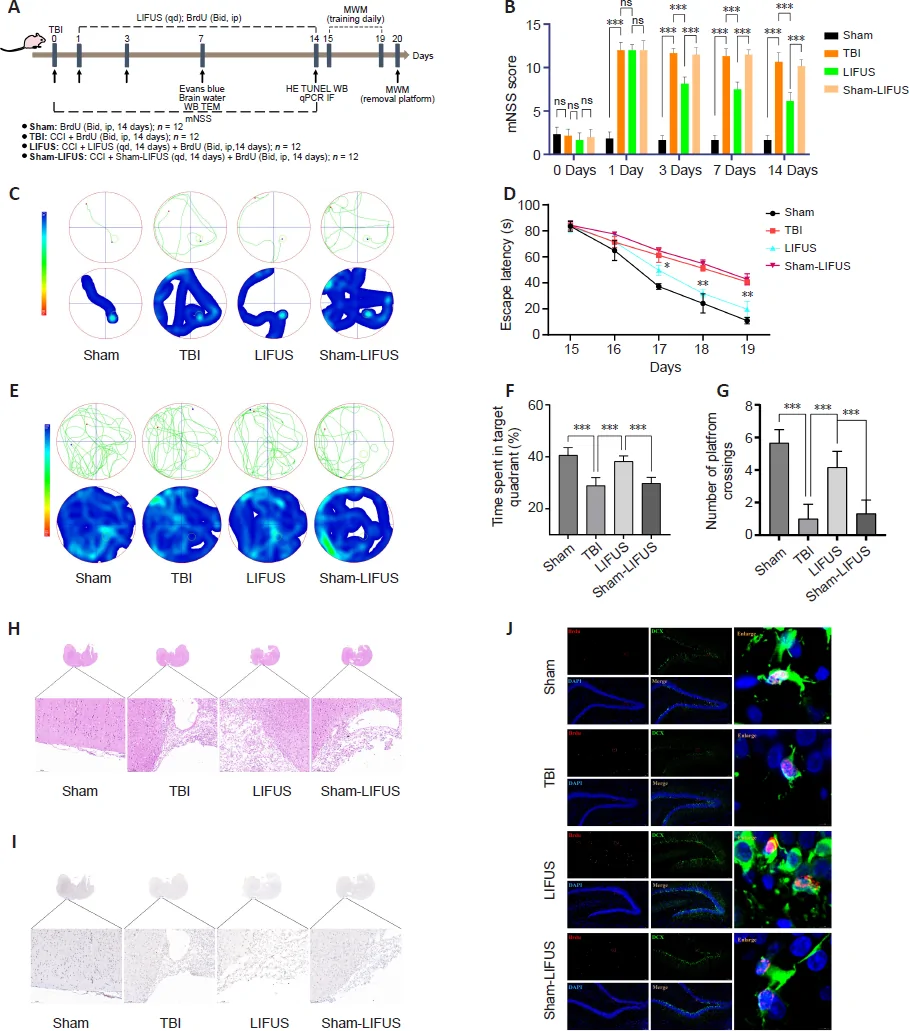
LIFUS promotes angiogenesis and neurogenesis after TBI. (A) Experimental timeline of the animal experiments. (B) mNSS at baseline and on days 1, 3, 7, and 14 post-TBI (*n* = 6). (C) Movement traces and heat maps from the MWM training phase. (D) MWM latency. (E) Movement traces and heat maps from the MWM testing phase. (F) Time spent in the target quadrant in the MWM. (G) Number of platform crossings in the MWM. (H) Representative images of HE-stained brain tissue (*n* = 3). Compared with the sham group, the TBI and sham-LIFUS groups showed sparse brain tissue structure with obvious vacuolar degeneration and death, while LIFUS treatment alleviated vacuolar degeneration and necrosis. Scale bar: 100 μm. (I) Immunohistochemical staining of brain tissue for CD31 (*n* = 3). Scale bar: 100 μm. (J) Representative images of DCX (green, iF488)/BrdU (red, iF555) double-staining of the hippocampus. Scale bars: 100 μm, 5 μm (enlarged image). Results are presented as mean ± SD. *P < 0.05, **P < 0.01, ***P < 0.001 (one-way analysis of variance followed by Tukey’s *post hoc* test). Bid: Bisindie; BrdU: bromodeoxyuridine; CCI: controlled cortical impact; DCX: doublecortex; DAPI: 4′,6-diamidino-2-phenylindole; EB: Evans blue; HE: hematoxylin-eosin staining; ip: intraperitoneal injections; IF: immunofluorescence; LIFUS: low-intensity focused ultrasound; mNSS: modified neurological severity scale; MWM: Morris water maze; qd: once a day; QPCR: quantitative polymerase chain reaction; TBI: traumatic brain injury; TUNEL: terminal dexynucleotidyl transferase-mediated dUTP nick end labeling; TEM: transmission electron microscopy; WB: Western blot.

To investigate whether LIFUS can promote angiogenesis and neurogenesis, as well as improve neurological function, we performed a series of animal experiments. The experimental timeline for these studies is illustrated in **Figure 1A**. The mNSS was determined at baseline and on post-TBI days 1, 3, 7, and 14 to assess the impact of LIFUS on neurological impairment (**Figure 1B**). Prior to TBI induction, there were no significant differences in mNSS scores among the experimental groups. On the first day post-TBI, before LIFUS intervention, the TBI group exhibited a marked increase in mNSS scores compared with the sham group, with no significant variations among the treatment groups. However, on post-TBI days 3, 7, and 14, the TBI group continued to show elevated scores in contrast to the sham group. Notably, LIFUS treatment significantly reduced mNSS scores during this period, while no decrease in scores was observed in the sham-LIFUS group, suggesting that LIFUS has neuroprotective effects against neural injury. Results from the Morris water maze experiment indicated a significant reduction in escape latency to the platform after 2 weeks of LIFUS treatment compared with both the TBI and sham-LIFUS groups. Additionally, a notable increase in the time spent in the target quadrant and the number of platform crossings was observed (**Figure 1C–G**). Together, these results indicate that LIFUS improved both cognitive and motor function in TBI rats. To evaluate the physiological basis of the therapeutic efficacy of LIFUS on TBI, HE staining was performed on brain tissue samples from each experimental group. The sham-operated group exhibited a well-defined, densely packed brain tissue structure with normal neuronal morphology. By contrast, both the TBI and sham-LIFUS groups displayed sparse tissue structures characterized by significant vacuolar degeneration and cellular death. Remarkably, LIFUS treatment significantly decreased the size and number of brain tissue lesions and reduced cell death, suggesting its therapeutic potential for TBI management (**Figure 1H**). Immunohistochemical analysis of brain tissue sections revealed a notable reduction in blood vessel density at the injury site in the TBI group compared with the sham group. Conversely, LIFUS treatment significantly increased the blood vessel count relative to the TBI group, while the sham-LIFUS group showed no significant changes (**Figure 1I** and **Additional Figure 2A**). Furthermore, the TBI group exhibited a significant reduction in expression of the hippocampal neurogenesis marker double cortex (DCX) and a marked increase in expression of the proliferation marker BrdU compared with the sham group. Following LIFUS treatment, there was a significant enhancement in DCX expression and a more pronounced increase in BrdU labeling compared with the TBI group. Additionally, there was a notable increase in the number of cells that were positive for both BrdU and DCX. The sham-LIFUS group displayed no significant changes compared with the TBI group (**Figure 1J** and **Additional Figure 2B–D**). These findings indicate that LIFUS can improve neurological function in TBI rats and promotes angiogenesis and neurogenesis.

### Low-intensity focused ultrasound enhances angiogenesis and neurogenesis and activates Orexin-A and Orexin-A receptor 1

**Figure 2:**
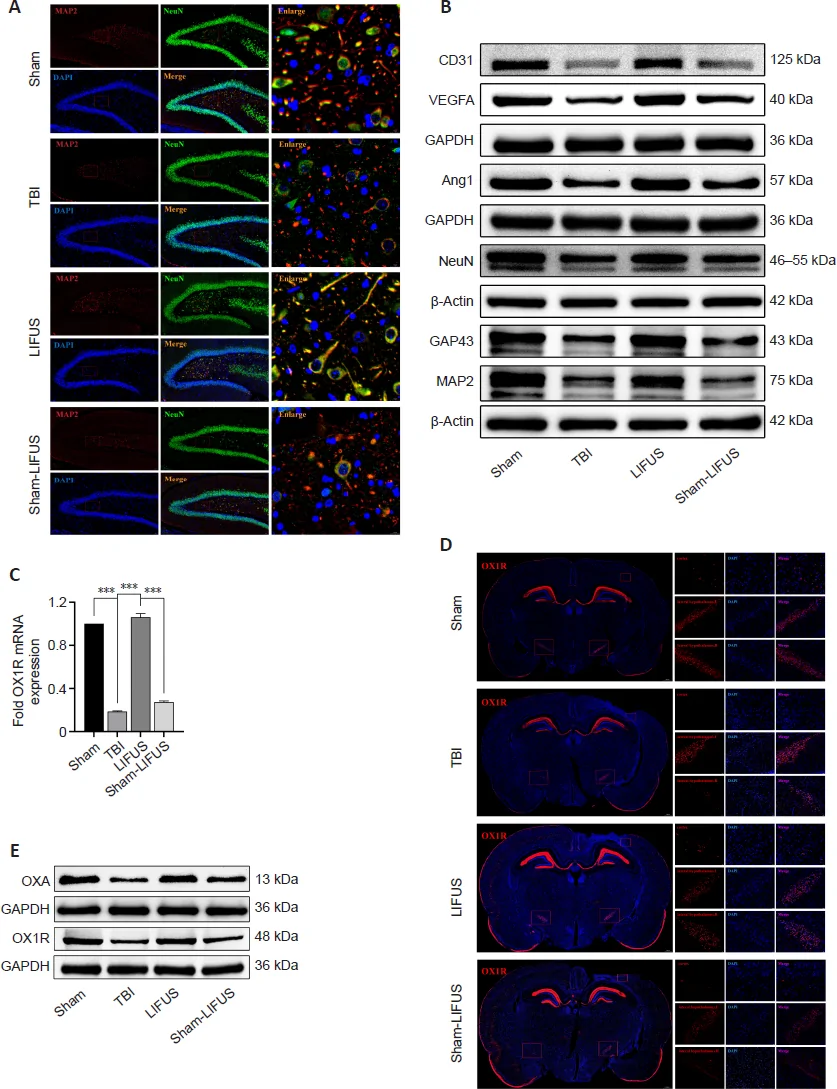
LIFUS enhances angiogenesis and neurogenesis and upregulates OXA and OX1R in the brain in a rat model of TBI rats. (A) Representative images of MAP2 (red, iF555)/NeuN (green, iF488) immunofluorescence double-staining of the hippocampus. Compared with the sham group, MAP2 expression in the hippocampus of rats in the TBI and sham-LIFUS groups was significantly decreased, while MAP2 expression was significantly increased after LIFUS treatment. The number of MAP2/NeuN double-labeled neurons was significantly increased in the LIFUS group compared with the TBI and sham-LIFUS groups. Scale bars: 50 μm, 10 μm (enlarged image). (B) Representative western blot images (*n* = 3). The quantitative results are shown in Additional Figure 2. (C) OX1R mRNA expression (*n* = 3). (D) Representative images of OX1R (red, iF555) immunofluorescence in brain tissue samples (*n* = 3). Scale bars: 500 μm, 50 μm (enlarged image). (E) Western blot analysis of OXA and OX1R expression (*n* = 3). The quantitative results are shown in Additional Figure 2. Results are presented as the mean ± SD. ***P < 0.001 (one-way analysis of variance followed by Tukey’s *post hoc* test). Ang1: Angiopoietin 1; DAPI: 4′,6-diamidino-2′-phenylindole; GAP43: growth-associated protein 43; GAPDH: glyceraldehyde-3-phosphate dehydrogenase; LIFUS: low-intensity focused ultrasound; MAP2: microtubule-associated protein 2; NeuN: neuronal nuclear antigen; OX1R: orexin receptor 1; PECAM-1/CD31: platelet endothelial cell adhesion molecule-1; TBI: traumatic brain injury; VEGFA: vascular endothelial growth factor A.

To further investigate whether LIFUS can enhance angiogenesis and neurogenesis, and to determine if these therapeutic effects are associated with OXA/OX1R, we conducted immunofluorescence staining and western blot analysis. The hippocampal region of rats from each group was subjected to immunofluorescence staining for the neurogenesis-related protein MAP2. Notably, there was a significant increase in the number of cells that were positive for both BrdU and DCX. MAP2 expression increased markedly following LIFUS treatment compared with the TBI group, resulting in a significant increase in the number of cells expressing both NeuN and MAP2. By contrast, the sham-LIFUS group exhibited no significant changes compared with the TBI group (**Figure 2A** and **Additional Figure 3A** and **B**). Western blot analysis (**Figure 2B** and **Additional Figure 3C–H**) demonstrated significant decreases in platelet endothelial cell adhesion molecule-1 (PECAM-1/CD31), angiopoietin 1 (Ang1), VEGFA, neuronal nuclear antigen (NeuN), growth-associated protein 43 (GAP43), and microtubule-associated protein 2 (MAP2) expression in injured brain tissue from the TBI group compared with the sham group. Conversely, LIFUS treatment significantly increased the expression levels of these proteins compared with the TBI group. No changes in protein expression were observed in the sham-LIFUS group. These findings highlight the beneficial effects of LIFUS in promoting recovery from TBI, particularly by enhancing angiogenesis and neurogenesis. Next, RNA was isolated from the injured rat brain tissue to evaluate OX1R mRNA expression levels (**Figure 2C**). The TBI group exhibited a significant reduction in OX1R mRNA expression compared with the sham surgery group. By contrast, the LIFUS group displayed a marked increase in OX1R mRNA expression, while the sham-LIFUS group showed significantly lower OX1R mRNA levels compared with the LIFUS group. Immunofluorescence analysis of OX1R was performed on whole-brain sections from rats, allowing for comparisons across different brain regions (**Figure 2D** and **Additional Figures 3I–K**). In the injured cortical area, the TBI group demonstrated a significant decrease in the number of OX1R-positive cells compared with the sham group, while the LIFUS-treated group exhibited a marked increase in the number of OX1R-positive cells. The sham-LIFUS group did not show a substantial increase in the number of OX1R-positive cells. No significant differences in OX1R expression were observed in the left lateral hypothalamus among the groups. However, in the right lateral hypothalamus (contralateral to the injury), OX1R expression was significantly reduced in the TBI group compared with the sham group, while it was notably elevated in the LIFUS group. The sham-LIFUS group did not exhibit significant changes compared with the TBI group. This finding suggests that LIFUS may enhance OX1R expression in the lateral hypothalamus. Western blot analysis (**Figure 2E** and **Additional Figure 3L** and **M**) showed a significant decrease in OXA and OX1R expression levels in the TBI group compared with the sham group. Conversely, the LIFUS-treated group showed a significant increase in both OXA and OX1R expression. Additionally, a notable decrease in OXA and OX1R levels was found in the sham-LIFUS group compared with the LIFUS-treated group. Overall, these results suggest that LIFUS promotes angiogenesis and neurogenesis, potentially through mechanisms closely related to OXA/OX1R signaling.

### Low-intensity focused ultrasound treatment reduces neuronal necrosis via Orexin-A

**Figure 3:**
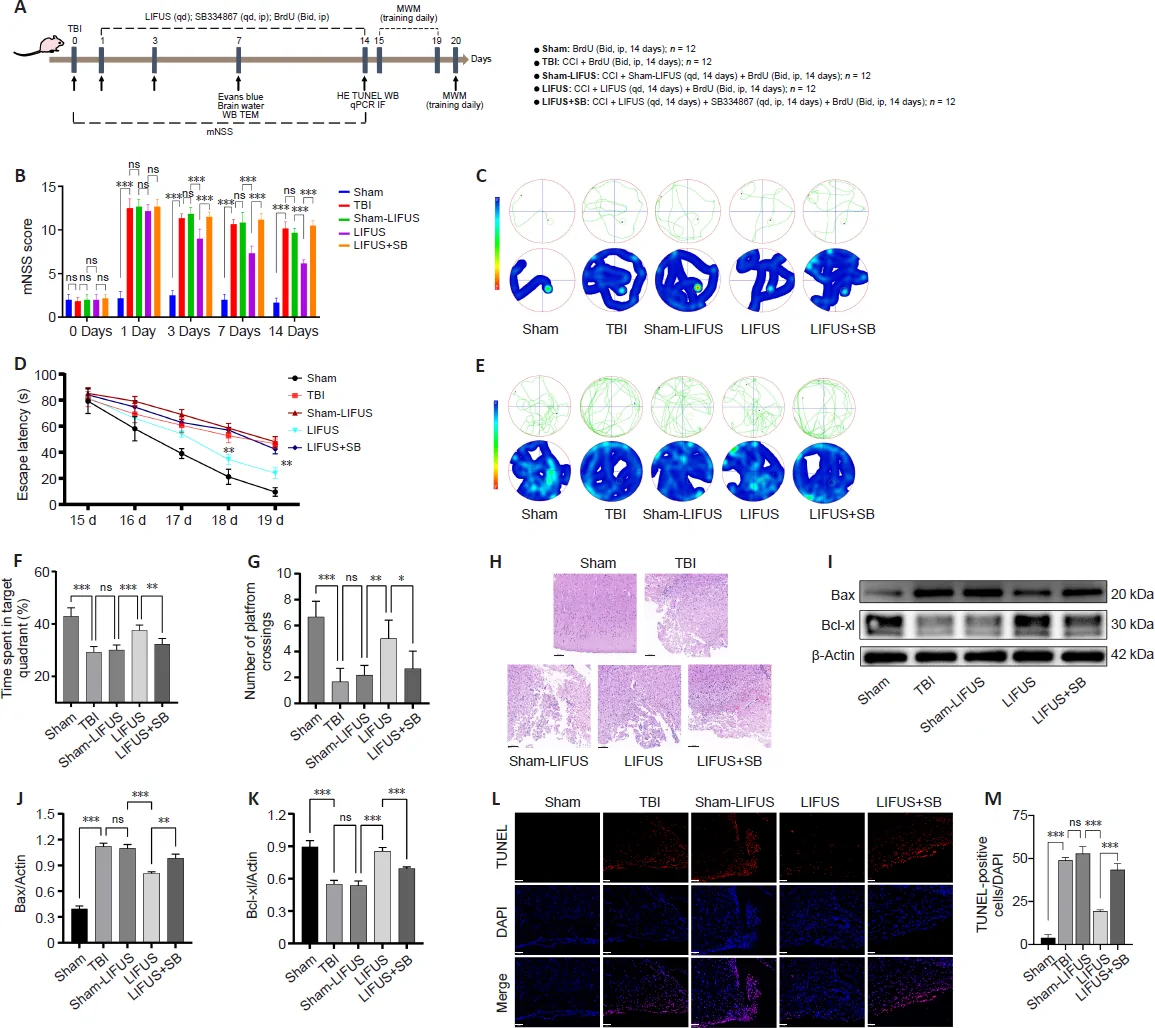
OXA is a key modulator of the LIFUS-induced reduction in neuronal necrosis in a rat model of TBI. (A) Experimental timeline of the animal experiments. (B) mNSS scores. (C) Movement traces and heat maps from the MWM training phase. (D) MWM latency in the training phase. (E) Movement traces and heat maps from the MWM testing phase. (F) Time spent in the target quadrant in the MWM testing phase. (G) Number of platform crossings in the MWM testing phase. (H) Representative images of HE-stained rat brain tissue. Compared with the sham group, the TBI group and sham-LIFUS group exhibited sparse brain tissue structure and obvious vacuolar degeneration, while LIFUS treatment reduced vacuolar degeneration, and SB334867 significantly inhibited the effects of LIFUS. Scale bar: 100 μm. (I) Western blot images analysis of Bax and Bcl-xl expression (*n* = 3). (J) Quantification of Bax expression in brain tissue. (K) Quantification of Bcl-xl expression in brain tissue. (L) Representative TUNEL staining images (*n* = 3). Scale bar: 50 μm. (M) Quantification of TUNEL-positive cells. Results are presented as the mean ± SD. *P < 0.05, **P < 0.01, ***P < 0.001 (one-way analysis of variance followed by Tukey’s *post hoc* test). Bid: Bisindie; Bax: Bcl-2 associated X protein; Bcl-xl: B-cell lymphoma-extra large; BrdU: bromodeoxyuridine; CCI: controlled cortical impact; DAPI:4′,6-diamidino-2′-phenylindole; EB: Evans blue; GAPDH: glyceraldehyde-3-phosphate dehydrogenase; HE: hematoxylin-eosin staining; IF: immunofluorescence; ip: intraperitoneal injection; LIFUS: low-intensity focused ultrasound; mNSS: modified neurological severity scale; MWM: Morris water maze; qd: Quaque die; QPCR: quantitative polymerase chain reaction; SB334867: orexin receptor 1 inhibitor; TBI: traumatic brain injury; TEM: transmission electron microscopy; WB: Western blot; TUNEL: terminal dexynucleotidyl transferase-mediated dUTP nick end labeling.

To investigate the role of OXA/OX1R in LIFUS treatment of TBI, we used SB334867, an OX1R inhibitor. The experimental timeline for these animal experiments is illustrated in **Figure 3A**. Neurological function was assessed by mNSS (**Figure 3B**). Prior to the induction of TBI, no significant differences in mNSS scores were observed among the groups. However, on the first day post-injury, just before LIFUS administration, the TBI group exhibited a significant increase in mNSS scores compared with the sham-operated group, while there were no notable variations among the different treatment groups at this time point. On days 3, 7, and 14 post-TBI, a significant reduction in mNSS scores was observed in response to LIFUS treatment. By contrast, administration of SB334867 (an OXA antagonist) significantly diminished this effect. Additionally, the results from the Morris water maze experiment indicated that SB334867 reversed the beneficial effects of LIFUS treatment (**Figure 3C–G**). Next, histological evaluation of brain tissue samples was conducted using hematoxylin and eosin (HE) staining (**Figure 3H**). The sham-operated group exhibited a well-preserved brain tissue architecture with normal neuronal distribution. Both the TBI and sham-LIFUS groups, however, exhibited sparse tissue structures characterized by significant vacuolar changes and cell death. Remarkably, LIFUS intervention effectively mitigated brain tissue damage and reduced cell death. Conversely, administration of SB334867 markedly counteracted these effects. Western blot analysis conducted during the second week post-injury (**Figure 3I–K**) revealed a significant increase in expression of the pro-apoptotic protein Bax and a notable decrease in expression of the anti-apoptotic protein Bcl-2 in the TBI group compared with the sham group. The sham-LIFUS group showed no significant differences from the TBI group. By contrast, LIFUS treatment resulted in a significant decrease in Bax expression and a notable increase in Bcl-2 expression. Importantly, SB334867 significantly inhibited the therapeutic effects of LIFUS. TUNEL staining (**Figure 3L** and **M**) showed an increased number of apoptotic cells in both the TBI and sham-LIFUS groups compared with the sham group. However, LIFUS treatment led to a significant reduction in the number of TUNEL-positive cells, and this effect was significantly reversed by administration of SB334867. These results suggest that OXA mediates the beneficial effects of LIFUS on neurological function in rats subjected to TBI.

### Orexin-A is indispensable for low-intensity focused ultrasound-mediated restoration of blood–brain barrier functionality in a rat model of traumatic brain injury

**Figure 4:**
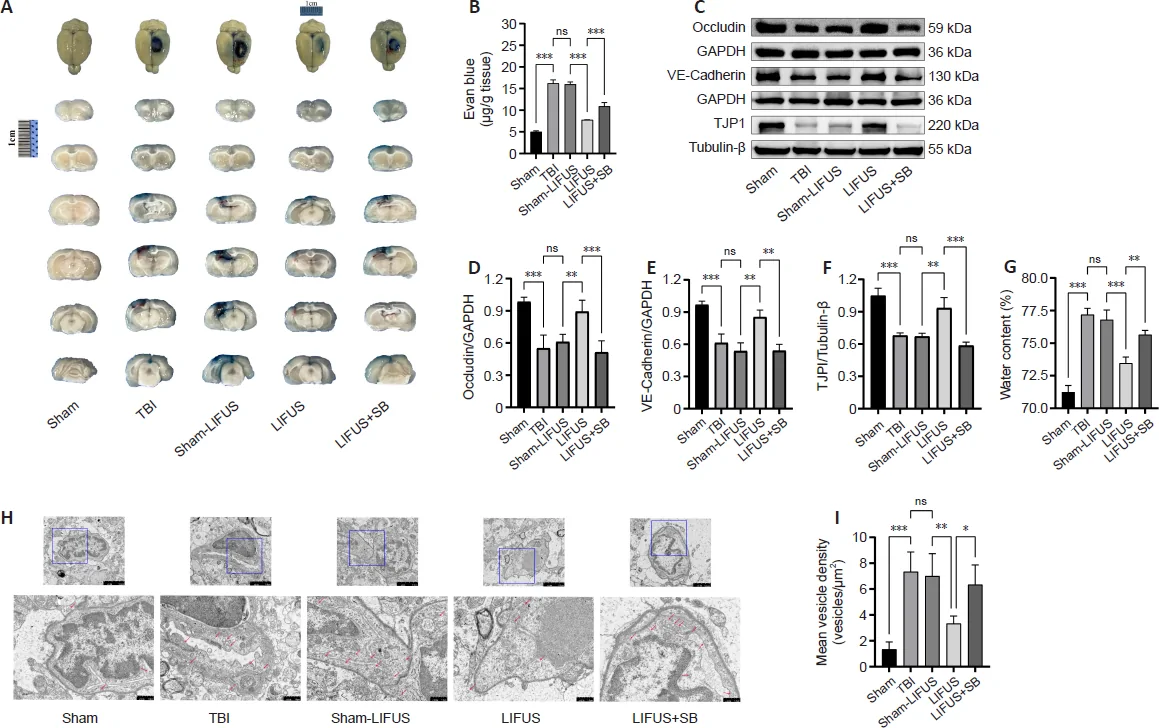
OXA is indispensable for mediating the LIFUS-induced restoration of blood–brain barrier function after traumatic brain injury in rats. (A) Representative whole-brain images and coronal sections illustrating Evans blue extravasation 3 days post-TBI (*n* = 3). Scale bar: 1 cm. (B) Quantitative analysis of Evans blue extravasation (*n* = 3). (C) Representative western blot images showing VE-Cadherin, TJP1, and Occludin expression (*n* = 3). (D–F) Quantification of Occludin, VE-Cadherin, and TJP1 expression in the brain. (G) Brain water content 7 days after TBI (*n* = 3). (H) Transmission electron microscopy images depicting caveolae-like vesicles in brain microvascular endothelial cells. Scale bars: 5 μm, 1 μm (for enlarged images). (I) Quantification of caveolae-like vesicle density. Results are presented as the mean ± SD. *P < 0.05, **P < 0.01, ***P < 0.001 (one-way analysis of variance followed by Tukey’s *post hoc* test). LIFUS: Low-intensity focused ultrasound; OXA: orexin-A; SB: SB334867; TBI: traumatic brain injury; TJP1: tight junction protein; VE-Cadherin: vascular endothelial Ca^2+^-dependent cell adhesion molecule family.

To further investigate the role of OXA in LIFUS-mediated protection of the blood–brain barrier (BBB), we conducted targeted studies of the BBB. We observed a significant increase in intracerebral Evans blue dye extravasation in the TBI group compared with the sham group, indicating enhanced BBB permeability after TBI. Notably, there was no difference in Evans blue dye extravasation between the TBI group and the sham-LIFUS group. By contrast, LIFUS treatment led to a significant reduction in Evans blue dye leakage. However, administration of SB334867 markedly diminished the therapeutic effectiveness of LIFUS (**Figure 4A** and **B**). Western blot analysis demonstrated a notable reduction in the levels of vascular endothelial Ca^2+^-dependent cell adhesion molecule (VE-cadherin), tight junction protein 1 (TJP1), and occludin in the TBI group compared with the sham group. Conversely, there were no changes in the expression of these proteins in the sham-LIFUS group compared with the TBI group. By contrast, LIFUS treatment resulted in a substantial restoration of VE-cadherin, TJP1, and occludin expression levels. The efficacy of LIFUS treatment was significantly compromised by treatment with SB334867 (**Figure 4C–F**). In addition, elevated levels of brain tissue hydration were observed in the TBI group compared with the sham group. While the sham-LIFUS group did not exhibit reduced post-traumatic brain edema, LIFUS treatment significantly decreased brain edema. Consistent with the findings described above, administration of SB334867 compromised the efficacy of LIFUS treatment (**Figure 4G**). Transmission electron microscopy imaging revealed a marked increase in vesicle density within brain microvascular endothelial cells from both the TBI and sham-LIFUS groups compared with the sham group. LIFUS treatment reduced this vesicle density, while SB334867 reversed this effect (**Figure 4H** and **I**). These results indicate that OXA mediates the BBB-protective effects of LIFUS in rats subjected to TBI.

### Low-intensity focused ultrasound enhances angiogenesis and neurogenesis via Orexin-A

**Figure 5:**
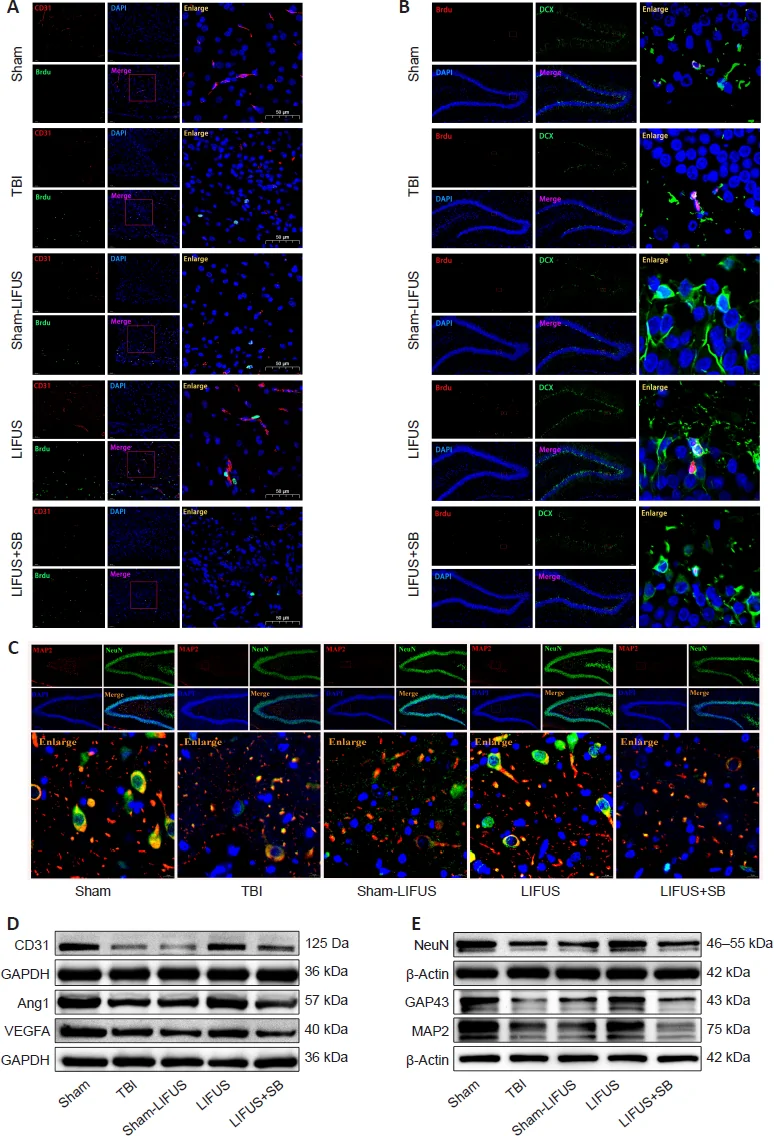
LIFUS enhances angiogenesis and neurogenesis via OXA. (A) Representative immunofluorescence images showing CD31 (red, iF555) and BrdU (green, iF488) double-positive cells in brain tissue (*n* = 3). Scale bar: 50 μm. (B) Representative immunofluorescence images depicting DCX (red) and BrdU (green) double-positive cells (*n* = 3). Scale bars: 100 μm, 5 μm (for enlarged images). (C) Representative immunofluorescence images illustrating co-expression of MAP2 (red, iF555) and NeuN (green, iF488) in double-positive cells (*n* = 3). Scale bars: 50 μm, 10 μm (for enlarged images). (D, E) Representative western blot images. Ang1: Angiopoietin 1; BrdU: bromodeoxyuridine; DCX: doublecortin; DAPI: 4′,6-diamidino-2-phenylindole; GAPDH: glyceraldehyde-3-phosphate dehydrogenase; GAP43: growth-associated protein 43; LIFUS: low-intensity focused ultrasound; MAP2: microtubule-associated protein 2; NeuN: neuronal nuclear antigen; OX1R: orexin receptor 1; OXA: orexin A; PECAM-1/CD31: platelet endothelial cell adhesion molecule-1; TBI: traumatic brain injury;VEGFA: vascular endothelial growth factor A.

To investigate the role of OXA in LIFUS-induced angiogenesis and neurogenesis, we conducted immunofluorescence staining of the ipsilateral hemisphere near the injury boundary (**Figure 5A** and **Additional Figure 4A–C**). Compared with the sham group, both the TBI and sham-LIFUS groups exhibited a significant decrease in the proportion of CD31-positive cells. By contrast, the LIFUS treatment group showed a notable increase in CD31-positive cells; however, this effect was significantly reversed following administration of SB334867. These findings highlight a substantial enhancement in microvessel density at the site of brain injury in TBI rats following LIFUS therapy. Quantitative analysis of BrdU staining revealed a significant increase in the proportion of BrdU-positive cells in both the TBI and sham-LIFUS groups compared with the sham group. Importantly, the LIFUS treatment group exhibited an even more pronounced increase in BrdU-positive cell abundance. However, the effects of LIFUS were significantly counteracted by SB334867. Additionally, we observed a marked increase in BrdU^+^/CD31^+^ cell counts in the LIFUS group compared with the TBI and sham-LIFUS groups, while the sham group showed minimal evidence of angiogenesis. Administration of SB334867 effectively reversed this enhancement. Double immunofluorescence staining for DCX and BrdU revealed a significant reduction in neurogenesis in the hippocampal region of TBI rats. By contrast, LIFUS treatment significantly improved neurogenesis in this area. However, SB334867 markedly inhibited LIFUS-induced neurogenesis (**Figure 5B** and **Additional Figure 4D–F**). Furthermore, double immunofluorescence staining for MAP2 and NeuN indicated a significant reduction in MAP2 expression in the hippocampal region of both the TBI and sham-LIFUS groups compared with the sham group, while LIFUS treatment led to a significant upregulation of MAP2 expression. Again, SB334867 notably inhibited the effects of LIFUS (**Figure 5C** and **Additional Figure 4G** and **H**). To further validate these findings, we performed western blot analysis to assess the expression levels of VEGFA, Ang1, CD31, NeuN, GAP43, and MAP2 in brain tissue adjacent to the injury site (**Figure 5D** and **E**, and **Additional Figure 4I–N**). The TBI group displayed reduced levels of these proteins compared with the sham group, and no significant difference was observed between the sham-LIFUS and TBI groups. LIFUS treatment restored the expression levels of these proteins, but this effect was significantly diminished by administration of SB334867. Overall, these results indicate that OXA plays a crucial role in promoting angiogenesis and neurogenesis following LIFUS treatment.

### Low-intensity focused ultrasound can activate the orexin-A–mitogen-activated protein kinase signaling pathway

**Figure 6:**
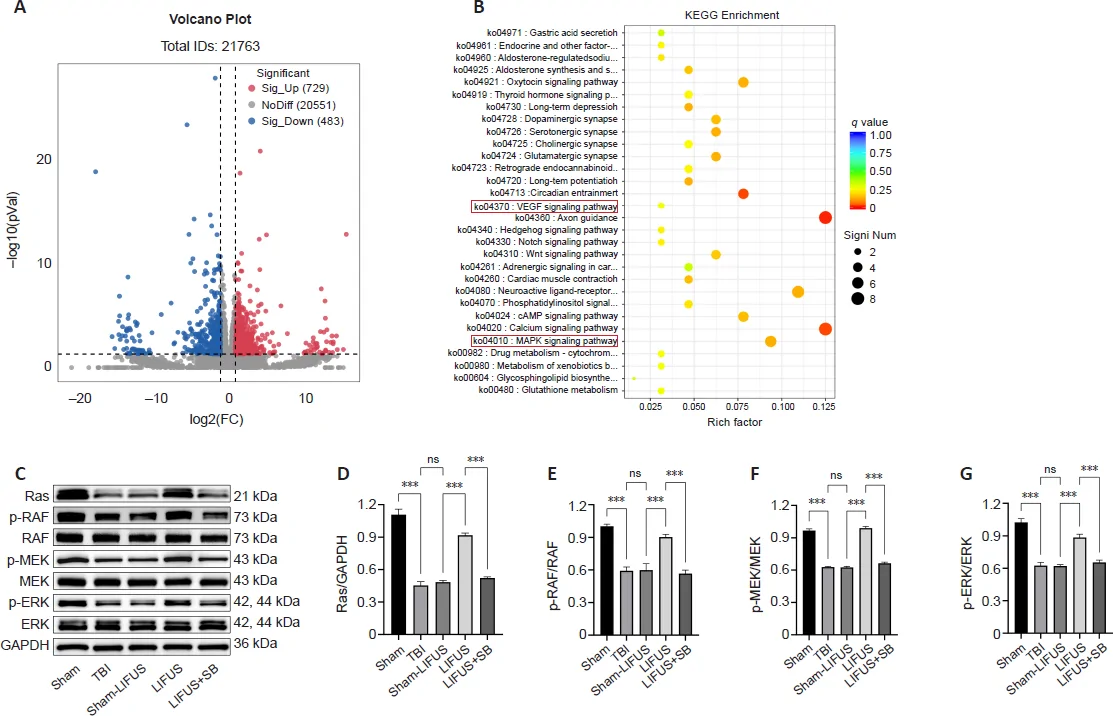
LIFUS promotes angiogenesis and neurogenesis after TBI through the OXA-MAPK signaling pathway. (A) Volcano plot of differentially expressed genes identified by transcriptome sequencing. (B) Bubble map of KEGG enrichment analysis. (C) Western blot analysis. Band densities were normalized to GAPDH as the internal reference (*n* = 3). (D–G) Quantification of Ras, p-RAF/RAF, p-MEK/MEK, and p-ERK/ERK expression (*n* = 3). Results are presented as the mean ± SD. ***P < 0.001 (one-way analysis of variance followed by Tukey’s *post hoc* test). ERK: Extracellular regulated protein kinases; LIFUS: low-intensity focused ultrasound; MAPK: mitogen-activated protein kinase; MEK: mitogen-activated extracellular signal regulated kinase; OXA: orexin-A; SB: SB334867; Ras: rat sarcoma; Raf: Raf protein kinase; TBI: traumatic brain injury.

To further investigate the mechanisms by which LIFUS promotes angiogenesis and neurogenesis after TBI, we performed transcriptome sequencing on brain tissue collected from the injury sites of rats in both the TBI and LIFUS treatment groups. Our analysis identified 1212 differentially expressed genes, of which 729 genes were upregulated and 483 were downregulated (**Figure 6A**). Kyoto Encyclopedia of Genes and Genomes (KEGG) enrichment analysis of the differentially expressed genes showed significant enrichment of several signaling pathways, including the mitogen-activated protein kinase (MAPK), VEGFA, Wnt, and cyclic adenosine monophosphate (cAMP) pathways (**Figure 6B**). The MAPK pathway is a critical signaling pathway that regulates both angiogenesis and neurogenesis (Kim et al., 2015; Huang et al., 2019; Pakdeepak et al., 2021; Sghaier et al., 2022; Taha et al., 2023). To determine whether the LIFUS-induced increase in OXA expression affects the MAPK pathway and promotes angiogenesis post-TBI, we assessed the expression levels of proteins within this signaling pathway by western blot. The TBI group demonstrated significantly reduced expression levels of Ras, p-Raf/Raf, p-MEK/MEK, and p-ERK/ERK proteins compared with the sham group. In the sham-LIFUS group, there were no significant changes in these protein levels compared with the TBI group, although some variations were noted. By contrast, LIFUS treatment significantly increased the levels of these proteins, and this response was notably attenuated by co-administration of SB334867 (**Figure 6C–G**). These findings suggest that LIFUS activates the MAPK signaling pathway via OXA.

### Orexin-A significantly mitigates tumor necrosis factor-α–induced cellular damage *in vitro*

**Figure 7:**
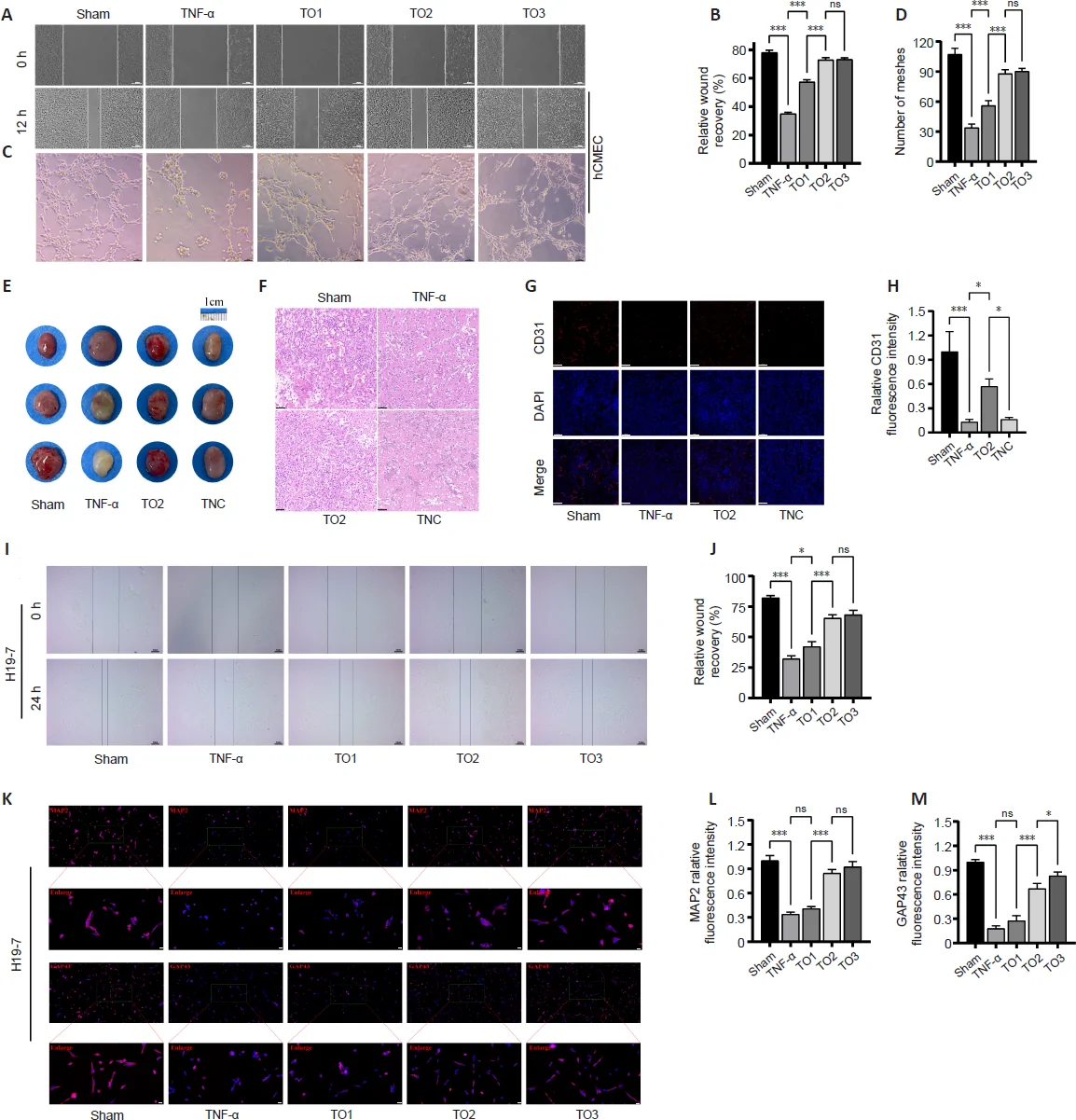
OXA significantly mitigates TNF-α–induced cellular damage *in vitro*. (A) The impact of OXA (0.1, 0.5, and 1 μM) on wound healing in hCMEC/D3 cells injured by TNF-α (10 ng/mL). TNF-α decreased HCMEC/D3 cell migration compared with the control group, while 0.1 or 0.5 μM OXA significantly improved wound healing. Conversely, elevated levels of OXA (1 μM) did not demonstrate a proportional enhancement in repair effectiveness. Scale bar: 200 μm. (B) Quantification of the effect of OXA on wound closure. (C) Representative images depicting the impact of 10 ng/mL TNF-α and three different concentrations (0.1, 0.5, and 1 μM) of OXA on tube formation. Scale bars: 40 μm. (D) The influence of OXA on capillary-like tube formation. (E) Representative images of Matrigel plugs. TNF-α significantly inhibited blood vessel formation in the Matrigel plug assay, and OXA alleviated this effect. Scale bar: 1 cm. (F) Representative images of Matrigel plugs stained with HE. Compared with the control group, the vessel density was significantly reduced in the TNF-α group and significantly increased in the TO2 group. Scale bars: 50 μm. (G) Representative images of Matrigel plugs subjected to CD31 immunofluorescence. Scale bar: 100 μm. (H) Quantification of CD31-positive cells (*n* = 3). (I) Wound healing assay in H19-7 cells injured with TNF-α (10 ng/mL) and treated with OXA at concentrations of 0.1 μM (TO1), 0.5 μM (TO2), and 1 μM (TO3). Compared with the control group, TNF-α treatment decreased cell migration capacity. However, pretreatment with 0.1 or 0.5 μM OXA significantly alleviated TNF-α-induced damage in H19-7 cells, whereas a higher OXA concentration (1 μM) did not significantly enhance migration compared with 0.5 μM OXA. Scale bar: 1 mm. (J) Quantification of the effect of OXA on wound closure (*n* = 3). (K) Representative images of the MAP2 (red, iF555) and GAP43 (red, iF555) immunofluorescence staining of H19-7 cells. Scale bars: 50 μm, 20 μm (enlarged images). (L) MAP2 fluorescence intensity in H19-7 cells. (M) GAP43 fluorescence intensity in H19-7 cells. Results are presented as means ± SD. *P < 0.05, ***P < 0.001 (one-way analysis of variance followed by Tukey’s *post hoc* test). TO1: TNF-α (10 ng/mL) + Orexin-A (0.1 μM); TO2: TNF-α (10 ng/mL) + Orexin-A (0.5 μM): TO3: TNF-α (10 ng/mL) + Orexin-A (1 μM); TNC: TNF-α (10 ng/mL) + normal saline. DAPI: 4′,6-Diamidino-2-phenylindole; GAP43: growth-associated protein 43; HE: hematoxylin-eosin staining; MAP2: microtubule-associated protein 2; OXA: orexin A; PECAM-1/CD31: platelet endothelial cell adhesion molecule-1; TNF-α: tumor necrosis factor-α.

To verify the effects of OXA on attenuating TNF-α-induced injury *in vitro* and to determine the optimal therapeutic concentration, we performed a wound healing assay to assess vascular endothelial cell (iCell-h070) and hippocampal neuron (H19-7) migration. TNF-α treatment reduced cell migration compared with the control group, while OXA at 0.1 and 0.5 μM significantly enhanced wound healing. However, higher concentrations of OXA (1 μM) did not show a proportional increase in repair effectiveness (**Figure 7A** and **B**). Next, we performed an *in vitro* capillary formation assay to assess the effects of OXA on tube formation. TNF-α treatment significantly suppressed tube formation by HCMEC/D3 cells compared with the control group. Notably, 0.1 and 0.5 μM OXA effectively restored the tube-forming capacity of HCMEC/D3 cells treated with TNF-α. By contrast, higher concentrations of OXA (1 μM) did not lead to any additional enhancement in tube formation (**Figure 7C** and **D**). On the basis of these results, we used 0.5 μM OXA in subsequent experiments investigating its potential mechanisms of action. *In vivo* Matrigel plug experiments were conducted to investigate the effects of OXA on vascular neogenesis. The results demonstrated that TNF-α significantly suppressed intravascular neogenesis, while OXA mitigated this effect (**Figure 7E**). The TNF-α group exhibited a notable decrease in vascular encirclements compared with the sham group, as indicated by HE staining of the Matrigel plugs. OXA administration resulted in a marked increase in the number of vascular encirclements (**Figure 7F**). Additionally, quantitative immunofluorescence analysis revealed a significant reduction in the mean fluorescence intensity of CD31-positive cells in the TNF-α group compared with the sham group. Conversely, treatment with OXA resulted in a substantial increase in the mean fluorescence intensity of CD31-positive cells (**Figure 7G** and **H**). To further investigate the ability of OXA to mitigate neural cell damage *in vitro*, we examined its effects on TNF-α–induced migration and the expression of growth-associated proteins in H19-7 rat hippocampal neuronal cells. A scratch assay was conducted to evaluate the effects of OXA on hippocampal neuronal cell migration (**Figure 7I** and **J**). TNF-α treatment decreased cell migration compared with the control group; however, pretreatment with 0.1 and 0.5 μM OXA significantly alleviated this effect. By contrast, a higher concentration of OXA (1 μM) did not significantly enhance migration compared with 0.5 μM OXA. Additionally, immunofluorescence analysis was conducted to assess the expression levels of growth-associated proteins across the different cell groups, thereby evaluating the role of OXA in promoting hippocampal neuron growth *in vitro* (**Figure 7K–M**). Compared with the control group, TNF-α treatment significantly inhibited MAP2 and GAP43 expression in H19-7 cells. Pretreatment with different concentrations of OXA led to a notable increase in the expression of both growth-associated proteins, with 0.5 μM OXA showing particularly significant effects. These results indicate that OXA significantly enhances vascular endothelial cell migration, tube formation, and blood vessel formation, while also promoting hippocampal neuron growth and migration.

### Orexin-A enhances angiogenesis and neurogenesis via the mitogen-activated protein kinase signaling pathway

**Figure 8:**
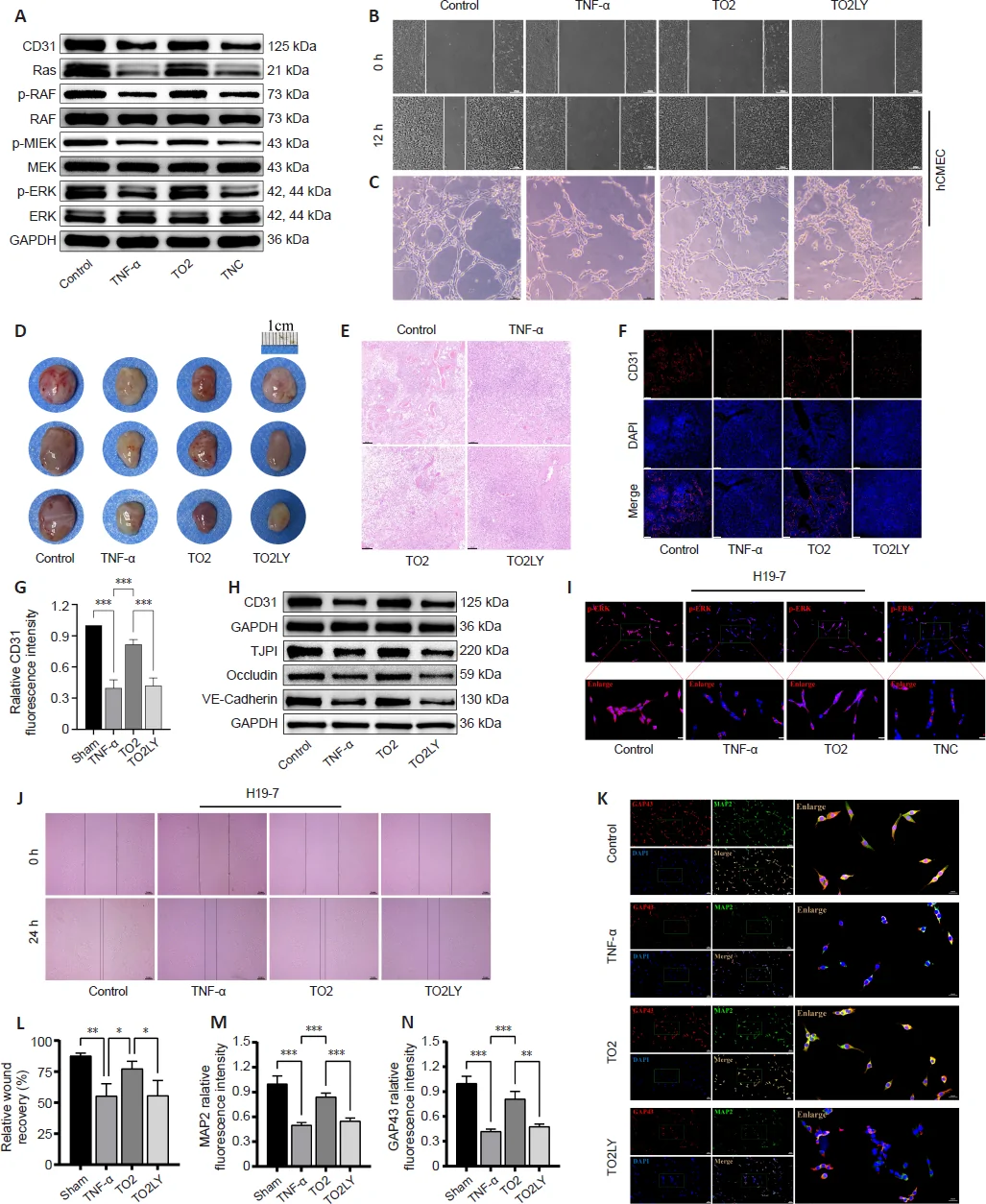
OXA enhances angiogenesis and neurogenesis via the MAPK pathway. (A) Representative western blot images. (B) The combined effects of LY3214996 (1 μM) and OXA (0.5 μM) on wound healing. Treatment with 0.5 μM OXA significantly enhanced endothelial cell migration following injury, while LY3214996 markedly reversed this effect. Scale bar: 200 μm. (C) The influence of concurrent application of LY3214996 (1 μM) and OXA (0.5 μM) on human cerebral microvascular endothelial cell (HCMEC) tube formation. LY3214996 reversed the ability of OXA to promote HCMEC tube formation. Scale bars: 40 μm. (D) Representative images of the Matrigel plugs used in the *in vivo* angiogenesis assay. Scale bars: 1 cm. (E) Representative images of the Matrigel plugs stained with hematoxylin and eosin (HE). Scale bars: 100 μm. (F) Representative images of Matrigel plugs subjected to CD31 immunofluorescence staining. Scale bar: 100 μm. (G) Quantitative analysis of CD31-positive cells (*n* = 3). (H) Western blot analysis of Matrigel plugs. (I) Representative images of p-ERK (red, iF555) immunofluorescence in H19-7 cells. Scale bars: 50 μm, 20 μm (for enlarged images). (J) Representative images illustrating the impact of co-treatment with LY3214996 (1 μM) and OXA (0.5 μM) on wound healing in H19-7 cells. Treatment with 0.5 μM OXA significantly enhanced the migration ability of H19-7 hippocampal neurons after TNF-α injury, while LY3214996 significantly reversed the effect of OXA. Scale bar: 1 mm. (K) Representative images showing MAP2 (green, iF488) and GAP43 (red, iF555) immunofluorescence in H19-7 cells. Scale bars: 100 μm, 20 μm (for enlarged images). (L) Quantification of scratch gap closure in each group. (M) MAP2 fluorescence intensity. (N) GAP43 fluorescence intensity. Results are presented as means ± SD. *P < 0.05, **P < 0.01, ***P < 0.001 (one-way analysis of variance followed by Tukey’s *post hoc* test). TNF-α: Tumor necrosis factor-α (10 ng/mL); TO2: TNF-α (10 ng/mL) + Orexin-A (0.5 μM); TO2LY: TNF-α (10 ng/mL) + Orexin-A (0.5 μM) + LY 3214996 (1 μM); TNC: TNF-α (10 ng/mL) + normal saline. DAPI: 4′,6-Diamidino-2-phenylindole; ERK: extracellular regulated protein kinases; GAP43: growth-associated protein 43; MAP2: microtubule-associated protein 2; MEK: mitogen-activated extracellular signal regulated kinase; PECAM-1/CD31: platelet endothelial cell adhesion molecule-1; Raf: Raf protein kinase; Ras: rat sarcoma.

To further investigate the mechanism by which OXA regulates the MAPK signaling pathway to promote angiogenesis and neurogenesis, we treated cells with the ERK1/2 inhibitor LY3214996. Western blot analysis of total protein from the extracellular matrix plug, showed a significant reduction in Ras, p-Raf, p-MEK, and p-ERK expression in the TBI group compared with the control group. However, administration of OXA led to a substantial increase in the levels of these proteins. By contrast, the solvent control TNC group showed no significant increase in protein expression (**Figure 8A** and **Additional Figure 5A–E**). To investigate the role of the MAPK pathway in OXA-mediated tube formation and neuronal growth, we conducted *in vitro* scratch and tube formation assays. The results indicated that 0.5 μM OXA significantly enhanced endothelial cell migration after injury, while LY3214996 reversed this effect (**Figure 8B** and **Additional Figure 5F**). Similarly, LY3214996 inhibited the ability of OXA to promote angiogenesis (**Figure 8C** and **Additional Figure 5G**). To further validate the role of OXA in promoting angiogenesis *in vivo* through the MAPK pathway, we used LY3214996 to inhibit ERK1/2 activity in a Matrigel plug assay. Gross images and HE staining of the Matrigel plugs showed that TNF-α significantly suppressed neovascularization, and this effect was alleviated by OXA. Moreover, simultaneous administration of TNF-α and the ERK1/2 inhibitor LY3214996 effectively restored the ability of OXA to promote angiogenesis (**Figure 8D** and **E**). Immunofluorescence analysis showed that the TNF-α group exhibited a marked decrease in the average fluorescence intensity of CD31-positive cells compared with the sham group. By contrast, OXA administration was associated with a noticeable increase in the mean fluorescence intensity of CD31-positive cells, an effect that was significantly inhibited by LY3214996 (**Figure 8F** and **G**). Additionally, the TNF-α group displayed significantly lower expression levels of CD31, TJP1, Occludin, and VE-Cadherin compared with the control group. Conversely, treatment with OXA resulted in marked upregulation of these proteins, while LY3214996 effectively countered the effects of OXA (**Figure 8H** and **Additional Figure 5H–K**). To further confirm that promotion of neural growth *in vitro* by OXA is related to the MAPK signaling pathway, we used detected p-ERK expression levels in H19-7 hippocampal neuronal cells across different groups via immunofluorescence (**Figure 8I** and **Additional Figure 5L**). TNF-α significantly reduced p-ERK expression levels, whereas OXA treatment led to a marked increase in p-ERK expression. The saline-treated control group showed no noticeable increase in p-ERK levels compared with the TNF-α group. A scratch assay indicated that LY3214996 significantly inhibited the OXA-induced cell migration (**Figure 8J** and **L**). Next, immunofluorescence double staining was used to assess expression levels of the neuronal growth proteins MAP2 and GAP43 in H19-7 cells from each group. TNF-α significantly decreased MAP2 and GAP43 expression, while OXA treatment restored the levels of these two neuronal growth proteins. However, LY3214996 inhibited the effects of OXA (**Figure 8K**, **M**, and **N**). These results suggest that OXA promotes both angiogenesis and neurogenesis by regulating the MAPK signaling pathway.

## Discussion

LIFUS for central nervous system disorders, particularly TBI, remains in the preclinical stage of laboratory investigation (Qi et al., 2024). The present study presents a groundbreaking finding: LIFUS enhances neurofunctional recovery in rats after brain injury by modulating the OXA-MAPK signaling pathway, which leads to increased angiogenesis and neurogenesis. Advancements in ultrasonic focusing and imaging technology have underscored the potential of LIFUS in regulating the central nervous system, generating significant scientific interest. A previous study has shown that stimulating the thalamic region in rats using LIFUS can increase extracellular levels of dopamine and serotonin while simultaneously reducing gamma-aminobutyric acid levels in the frontal lobe (Yang et al., 2012). The application of LIFUS has been proven to enhance brain-derived neurotrophic factor expression, thereby lessening the damage associated with TBI. Furthermore, LIFUS reduces BBB damage and brain edema, while providing neuroprotection (Su et al., 2017a, b). In our previous study, we found that LIFUS treatment of rats with acute TBI increased OXA expression, demonstrating an anti-neuroinflammatory effect (Huang et al., 2022). However, despite these advancements, the precise mechanism of action of LIFUS in TBI treatment remains incompletely understood. Therefore, the aim of the present study was to investigate the effects of LIFUS on neurogenesis and angiogenesis in a rat model of TBI. We observed a significant decrease in vascular density and hippocampal neurogenesis close to the TBI site, consistent with prior research (Watanabe et al., 2013; Wu et al., 2020; Guo et al., 2021). The reduction in the number of DCX-positive cells following TBI may have resulted from a combination of increased cell death and decreased cell division. After 2 weeks of LIFUS treatment, the newly generated blood vessels at the injury site had significantly increased, and neurogenesis in the hippocampal region was markedly enhanced. Notably, this was accompanied by a significant improvement in neurobehavioral function in TBI rats. Importantly, the OXA receptor antagonist SB334867 diminished this effect, indicating that OXA plays a crucial role in facilitating LIFUS-induced angiogenesis and neurogenesis, thereby enhancing neurological function in rats with TBI.

Vascular neogenesis is subject to an intricate regulatory system governed by a diverse array of angiogenic growth factors and regulators. Among these, VEGFA has emerged as a critical and indispensable regulator (Salehi et al., 2018; Guo et al., 2021; Wu et al., 2024). We found that VEGFA expression was reduced at the injury site on day 14 post-TBI, which contradicts findings from a prior study (Salehi et al., 2018). This discrepancy may be attributable to variations in the severity of TBI between the studies, differences in sample extraction sites, and variations in the timing of detection and assessment.

Neural repair is regulated by various neurotrophic factors and regulatory proteins, among which MAP2 and GAP43 are critical (Xiao et al., 2023; You et al., 2024). Elevated MAP2 and GAP43 expression levels are associated with neurorepair and regeneration in central nervous system injuries, including TBI, spinal cord injury, and ischemic stroke, establishing their role as biomarkers for neurogenesis (You et al., 2024). In our study, MAP2 and GAP43 expression levels in the cortical region surrounding the injury were markedly decreased in the TBI group compared with the sham group, consistent with the literature (Hood et al., 2018). LIFUS treatment significantly upregulated the expression of both proteins; however, this effect was inhibited by SB334867. These findings suggest that OXA regulates LIFUS-mediated neurogenesis.

Persistent BBB dysfunction following TBI is well documented (Jin et al., 2025). BBB disruption occurs rapidly after brain injury and can persist for extended periods, often correlating with unfavorable outcomes (Hay et al., 2015). In our study, we observed significant BBB disruption following TBI, as evidenced by structural alterations, pronounced cerebral edema, and decreased levels of key brain microvascular tight junction proteins (ZO-1, Occludin, and VE-Cadherin). Two weeks of LIFUS treatment notably attenuated TBI-induced BBB damage, as demonstrated by reduced Evans blue extravasation and a significant decrease in brain edema, which were associated with improved neurological function. These findings suggest that LIFUS may effectively reduce BBB disruption and brain edema resulting from TBI, in an OXA-dependent manner.

OXA has been recognized as a significant angiogenic factor that promotes blood vessel formation through activation of the MEK/ERK1/2 signaling pathway via OXA receptor stimulation (Kim et al., 2015), which aligns with the outcomes of the current study. In this study, we investigated the influence of OXA on HCMEC/D3 cell migration and tube formation through *in vitro* and *in vivo* Matrigel plug experiments. Our findings confirmed that OXA promotes endothelial cell migration and vascular formation via the OX1R-MAPK pathway. Additionally, *in vitro* experiments using hippocampal neuronal cells demonstrated that OXA plays a role in facilitating neuronal growth, specifically enhancing the growth of H19-7 hippocampal neuronal cells by modulating the MAPK signaling pathway. Importantly, the regulatory mechanisms of OXA extend beyond the MAPK signaling pathway. We previously showed that OXA influences the NF-κB/nucleotide-binding oligomerization domain, leucine-rich repeat and pyrin domain-containing 3 signaling pathway to exert anti-inflammatory effects (Huang et al., 2022). Furthermore, OXA regulates the nuclear factor erythroid 2-related factor 2/heme oxygenase 1 signaling pathway to exert anti-ferroptosis effects (Kang et al., 2024) and may also modulate the tissue factor/protein kinase B (AKT)/ERK signaling pathway to influence angiogenesis (Ren et al., 2024). Thus, OXA regulates multiple signaling pathways, and its *in vivo* effects likely involve various control and feedback loops. The specific details of these mechanisms remain unclear at present and will be the focus of future investigations.

This study has some limitations. First, because of species differences, rats cannot fully replicate human TBI. Second, while the Morris water maze test is widely used for assessing cognitive function, its relevance to complex neural function in humans may be limited. Future research should investigate the effects of LIFUS treatment through cross-species studies or clinical trials. Third, LIFUS was only used to treat moderate brain injury in male rats in this study; further research is needed to evaluate its therapeutic potential across different severities of brain injury and in female rats. Finally, our study focused solely on the effects of LIFUS treatment over a 2-week period, indicating the necessity for additional investigation into its long-term effects on neurological function in TBI rats.

In summary, our *in vivo* and *in vitro* findings indicate that LIFUS promotes angiogenesis and neurogenesis after TBI by activating the OXA-MAPK signaling pathway. In future studies, we will further investigate the use of LIFUS for TBI treatment to enhance its clinical applicability and precision.

## Additional files:

***Additional Figure 1:*** *Flowchart of animal experiments and cell experiments in this study.*

Additional Figure 1 Flowchart of animal experiments and cell experiments in this study.
(A) The specific procedure of animal experiments for LIFUS treatment of TBI; (B) The specific procedure of experiments for Matrigel plug assay; (C) detailed experimental flowchart of HCMEC/D3 cells; (D) detailed experimental flowchart of H19-7 cells. TNF-α: tumor necrosis factor-α (10 ng/mL); TO2: TNF-α (10 ng/mL) + Orexin-A (0.5 μM); TO2LY: TNF-α (10 ng/mL) + Orexin-A (0.5 μM) + LY3214996 (1 μM); TNC: TNF-α (10 ng/mL) + normal saline. CCI: Controlled cortical impact; HE: hematoxylin-eosin staining; IHC: immunohistochemistry; IF: immunofluorescence; LIFUS: low-intensity focused ultrasound; mNSS: modified neurological severity scale; MWM: Morris water maze; qPCR: quantitative polymerase chain reaction; TEM: transmission electron microscopy; WB: Western blot.

***Additional Figure 2:*** *LIFUS promotes angiogenesis and neurogenesis in TBI rats.*

Additional Figure 2 LIFUS promotes angiogenesis and neurogenesis in TBI rats.
(A) Intensity of CD31 expression in the injured brain, as shown in Figure 1I. (B) Fluorescence intensity of DCX in the injured brain. The data in panels A and B were normalized to the sham group. (C) Quantification of BrdU-positive cells in the injured brain. (D) Quantification of BrdU/DCX double-positive cells in the injured brain, as presented in Figure 1J. Results are expressed as the mean ± SD. ^*^P < 0.05, ^**^P < 0.01, ^***^P < 0.001 (one-way analysis of variance followed by Tukey's *post hoc* test). BrdU: Bromodeoxyuridine; DCX: doublecortin; DAPI: 4',6-diamidino-2-phenylindole; EB: Evans blue; LIFUS: low-intensity focused ultrasound; PECAM-1/CD31: platelet endothelial cell adhesion molecule-1; TBI, traumatic brain injury.

***Additional Figure 3:*** *LIFUS enhances angiogenesis and neurogenesis closely associated with OXA.*

Additional Figure 3 LIFUS enhances angiogenesis and neurogenesis closely associated with OXA.
(A) Quantification of microtubule-associated protein 2 (MAP2) expression in brain tissue. (B) Quantification of NeuN/MAP2 double-positive cells in brain tissue. (C-H) Quantification of expression levels of CD31, Ang1, VEGFA, NeuN, GAP43, and MAP2, as shown in Figure 2B (*n* = 3). (I-K) Quantification of OX1R expression fluorescence intensity in the cortex, left lateral hypothalamus, and right lateral hypothalamus, as depicted in Figure 2D. (L, M) Quantification of Orexin-A and OX1R levels in Figure 2E. Results are presented as means ± SD. ^*^P < 0.05, ^**^P < 0.01, ^***^P < 0.001 (one-way analysis of variance followed by Tukey's post hoc test). Ang1: Angiopoietin 1; GAPDH: glyceraldehyde-3-phosphate dehydrogenase; GAP43: growth-associated protein 43; LIFUS: low-intensity focused ultrasound; MAP2: microtubule-associated protein 2; NeuN: neuronal nuclear antigen; OX1R: orexin receptor 1; OXA: orexin A; TBI: traumatic brain injury; PECAM-1/CD31: platelet endothelial cell adhesion molecule-1; VEGFA: vascular endothelial growth factor A.

***Additional Figure 4:*** *OXA mediates LIFUS-induced angiogenesis and neurogenesis.*

Additional Figure 4 OXA mediates LIFUS-induced angiogenesis and neurogenesis.
(A) Rate of CD31-positive cells in Figure 5A. (B) Number of BrdU-positive cells in Figure 5A. (C) Number of CD31 and BrdU double-positive cells in Figure 5A. (D) Fluorescence intensity of doublecortin (DCX) expression in Figure 5B. (E) Number of BrdU-positive cells in Figure 5B. (F) Number of DCX and BrdU double-positive cells in Figure 5B. (G) Fluorescence intensity of microtubule-associated protein 2 (MAP2) expression in Figure 5C. (H) Quantification of the number of MAP2 and neuronal nuclear antigen (NeuN) double-positive cells in Figure 5C. (I-K) Quantification of expression levels of CD31, angiopoietin 1 (Ang1), and vascular endothelial growth factor A (VEGFA) in Figure 5D. (L-N) Evaluation of NeuN, growth-associated protein 43 (GAP43), and MAP2 expression in Figure 5E. Results are presented as the mean ± SD. ^*^P < 0.05, ^**^P < 0.01, ^***^P < 0.001 (one-way analysis of variance followed by Tukey's *post hoc* test). Ang1: Angiopoietin 1; BrdU: bromodeoxyuridine; DCX: doublecortin; DAPI: 4',6-diamidino-2-phenylindole; GAPDH: glyceraldehyde-3-phosphate dehydrogenase; GAP43: growth-associated protein 43; LIFUS: low-intensity focused ultrasound; MAP2: microtubule-associated protein 2; NeuN: neuronal nuclear antigen; OX1R: orexin receptor 1; OXA: orexin A; TBI: traumatic brain injury; PECAM-1/CD31: platelet endothelial cell adhesion molecule-1; VEGFA: vascular endothelial growth factor A.

***Additional Figure 5:*** *OXA promotes vascular formation in HCMEC/D3 cells and the growth of H19-7 neuronal cells, both of which are closely associated with the MAPK signaling pathway.*

Additional Figure 5 Autophagy-lysosome and mTORC2 signaling pathways in response to Rictor knockdown.
(A) Expression of P62, LC3A/B, RIPK1, and p-MLKL in shC-NSCs and shRictor-NSCs after 24 hours of TL and TL + CQ treatment. GAPDH was used as a loading control. (B) Expression of AKT and p-AKT in shC-NSCs and shRictor-NSCs after 24 hours of TL and TL + SC79 treatment. GAPDH was used as a loading control. Akt: Protein kinase B; CQ: chloroquine; GAPDH: glyceraldehyde-3-phosphate-dehydrogenase; LC3A/B: microtubule-associated protein 1 light chain 3 A/B; LPS: lipopolysaccharide; mTORC2: mammalian target of rapamycin complex 2; NSC: neural stem cell; p-Akt: phospho-protein kinase B; pMLKL: phospho-mixed lineage kinase domain-like protein; RIPK1: receptor interacting protein kinase 1; TL: TNF-α + LPS; TNF-α: tumor necrosis factor-alpha.

## Data Availability

*All data relevant to the study are included in the article or uploaded as Additional files.*
